# Cyclin-Dependent Kinase 5 Regulates cPLA2 Activity and Neuroinflammation in Parkinson’s Disease

**DOI:** 10.1523/ENEURO.0180-22.2022

**Published:** 2022-11-22

**Authors:** Sangita Paul, Saman Fatihi, Srishti Sharma, Rintu Kutum, Raymond Fields, Harish C. Pant, Lipi Thukral, Binukumar BK

**Affiliations:** 1 Council of Scientific and Industrial Research–Institute of Genomics and Integrative Biology, New Delhi 110007, India; 2 Academy of Scientific and Innovative Research (AcSIR), Ghaziabad 201002, India; 3 Viral Production Core Facility, National Institute of Neurological Disorders and Stroke, National Institutes of Health, Bethesda, Maryland 20892; 4 Neuronal Cytoskeletal Protein Regulation Section, National Institute of Neurological Disorders and Stroke, National Institutes of Health, Bethesda, Maryland 20892

**Keywords:** Cdk5, cPLA2, neuroinflammation, Parkinson’s disease

## Abstract

Hyperactivation of cyclin-dependent kinase 5 (Cdk5) by p25, contributes to neuroinflammation causing neurodegeneration in Parkinson’s disease (PD) and Alzheimer’s disease. However, the mechanism by which Cdk5 induces neuroinflammation in the PD brain is largely unexplored. Here, we show that Cdk5 phosphorylates cytosolic phospholipase A2 (cPLA2) at Thr-268 and Ser-505 sites lead to its activation and generation of eicosanoid products. Mutational studies using site-directed mutagenesis and molecular simulations show that the architecture of the protein changes on each single-point mutation. Interestingly, double mutations also led to a severe decline in the activity of cPLA2 and to the disruption of its translocation to the plasma membrane. Further, the brain lysates of transgenic PD mouse models show hyperactivation of Cdk5, resulting in enhanced phosphorylation of Thr-268 and Ser-505 of cPLA2 and its heightened activity, confirming the findings observed in the cell culture model of PD. These phosphorylation sites of cPLA2 and Cdk5 could be explored as the future therapeutic targets against neuroinflammation in PD. Further, conjoint transcriptomic analysis of the publicly available human PD datasets strengthens the hypothesis that genes of the arachidonic acid, prostaglandin synthesis, and inflammatory pathways are significantly upregulated in the case of PD patients compared with that of healthy control subjects.

## Significance Statement

Activated cytosolic phospholipase A2 (cPLA2) is a hallmark of neuroinflammation and is known to be hyperactive in Parkinson’s disease. Our study shows that Cdk5, which is also hyperactivated in neuroinflammation can phosphorylate cPLA2 at its novel site T-268A along with its known phosphorylation site S-505A. Both the sites of cPLA2 are shown to be critical for its activity. Also, mutation in both of the phosphorylation sites shows inhibition of cPLA2 membrane translocation and, therefore, decreases neuroinflammation. This provides a new insight for targeting neuroinflammation caused by hyperactive cPLA2 and Cdk5 in the case of PD.

## Introduction

Parkinson’s disease (PD) is one of the most commonly occurring neurodegenerative movement disorders worldwide (Allnutt et al., 2020). It is characterized by a progressive dopaminergic neuronal loss in the substantia nigra forming Lewy bodies (Dauer and Przedborski, 2003). Neurodegeneration is shown to have a strong link with microglial activation and chronic neuroinflammation in the PD brain (McGeer et al., 1988; Gao et al., 2002). Previous studies have reported the dysregulation of Cdk5 and increased p25/p35 ratio in the animal model of PD as well as in the brains of PD patients (Smith et al., 2003; Alvira et al., 2008; He et al., 2018), along with enhanced neuroinflammation (Surendranathan et al., 2015; Kouli et al., 2020). Neuroinflammation has also previously been considered to be a key player in PD pathogenesis (Qian et al., 2010); however, the precise mechanism behind the link remains unexplained.

Phospholipase A2 (PLA2) is an enzyme that catalyzes the hydrolysis of membrane phospholipids from the sn-2 position generating fatty acids such as arachidonic acid (AA) and lysophospholipids. Both molecules are potent inflammatory mediators resulting in immune response (Tan et al., 2016). PLA2 is classified into the following three groups: secretory PLA2 (sPLA2); cytosolic PLA2 (cPLA2); and Ca^2+^ independent PLA2. The cPLA2 or group IV PLA2 have four paralogs known in mammalian cells cPLA2-α, cPLA2-β, cPLA2-γ, and cPLA2-δ, of which cPLA2-α is the one most ubiquitously expressed (Kudo and Murakami, 2002; Litalien and Beaulieu, 2011). PLA2 is a requisite component in the cascade of events leading to the production of eicosanoids during acute and chronic inflammation. Moreover, the prolonged or unmodulated cPLA2 activation leads to membrane deacylation, excessive generation of eicosanoids, and uncontrolled Ca^2+^ influx. These effects ultimately result in lethal cellular injury (Stephenson et al., 1996). Previous evidence implicates inflammatory and immune mechanisms in the pathogenesis of PD and Alzheimer’s disease (Rogers et al., 2007). There is suggestive evidence that the treatment of PD patients with anti-inflammatory agents may be clinically beneficial (Bartels and Leenders, 2010; Thakur and Nehru, 2015).

Hence, the present study is focused to understand whether Cdk5 phosphorylates cPLA2, and, second, to determine whether phosphorylation of cPLA2 has any effect on its kinase activity as well as translocation to the plasma membrane. The functional significance of Cdk5/p25 on cPLA2 activation in primary astrocytes and glia/neuronal culture exposed to MPP^+^ was also explored. Our studies demonstrate the relevance of Cdk5-mediated phosphorylation of cPLA2 in the brain of transgenic PD mouse model where activated glia play a significant role leading to neuroinflammation. Finally, transcriptome analysis of human PD brain dataset compared with its control was performed to study the expression patterns of the genes involved in arachidonic acid and prostaglandin synthesis inflammatory pathways.

## Materials and Methods

### Animal handling

All animal experiments were performed according to the approved protocols by the Institutional Animal Care and Use Committee of the Council of Scientific and Industrial Research-Institute of Genomics and Integrative Biology of India. The PD transgenic mouse model Tg(Th-SNCA*A30P*A53T)39Eric and respective control mice were procured from the Centre for Cellular and Molecular Biology (Hyderabad, India). A maximum of six mice per cage were housed in a microisolator system and fed with a chow diet. There were 10 each control and PD transgenic mice. The PD group has six male and four female mice, and the control group has seven male and three female mice. The mice were killed at 7 months of age, and brains were properly stored for both protein extraction at −80° and sectioning in a 4% formaldehyde solution.

### Starting structure

The protein structure of human cPLA2 was taken from the RCSB (Research Collaboratory for Structural Bioinformatics) Protein Data Bank (PDB) Database (PDB ID: 1CJY). The missing residues in the crystal structure present in the C2 and the catalytic domain were modeled using I-TASSER (Iterative Threading Assembly Refinement; Yang et al., 2015). The point mutations for the residues T268 and S505 were generated with ChimeraX (Goddard et al., 2018).

### Molecular dynamics simulations

We performed all-atom molecular dynamics (MD) simulations using GROMACS 2018 (Lemkul, 2019) and the CHARMM36 force field (Huang et al., 2017). The water molecules were modeled with the TIP3P model. Periodic boundary conditions were used, and long-range electrostatic interactions were treated with Particle Mesh Ewald (Darden et al., 1993) summation using grid spacing of 0.16 nm combined with a fourth-order cubic interpolation to deduce the potential in between grid points. The real-space cutoff distance and van der Waals cutoff was set to 1.4 nm. The bond lengths were fixed, and a time step of 2 fs for numerical integration of the equation of motions was used (Hess et al., 1997). Coordinates were saved every 20 ps. Four independent MD trajectories, each 1 μs long at 310 K were conducted for cPLA2 wild-type (wt) and mutant structures. The four systems are referred to as wt, T268A, S505A, and double mutant (DM). The protein was placed in a cubic water box with a minimum of 1.0 nm of solvent on all sides. The systems were subjected to energy minimization using the steepest descent method. The simulations were subjected to a Nosé–Hoover T-coupling bath to maintain the exact temperature (Nosé, 1984). The structures were then subjected to Parrinello–Rahman barostat for pressure coupling at 1 bar (Parrinello and Rahman, 1981).

### Analysis of trajectories

The trajectories were analyzed for comparative structural changes using the GROMACS analysis toolkit. The secondary structural changes were calculated by the DSSP (hydrogen bond estimation algorithm) method (Frishman and Argos, 1995). Further, to understand intramolecular interactions, we used a network approach to study quantitative changes occurring near the mutant site across trajectories. The nodes represent the residues, and the interactions between residues are edges of the network. A cutoff distance of 0.5 nm was used to define a contact. All molecular images were generated using VMD (Visual Molecular Dynamics; Humphrey et al., 1996) and ChimeraX (Goddard et al., 2018) .

### Cloning of cPLA2 gene

The plasmid pcDNA3.1-CPLA2, which contains the human cPLA2 gene, was obtained from Li-Yuan Chen (National Institutes of Health/Clinical Center (CC)/Clinical Care Medicine Department (CCMD)). pcDNA3.1-CPLA2 was digested with EcoRI plus EcoRV and a 236 bp linker (GAATTCTGGATTGTGCTACCTACGTTGCTGGTCTTTCTGGCTCCACCTGGTATATGTCAACCTTGTATTCTCACCCTGATTTTCCAGAGAAAGGGCCAGAGGAGATTAATGAAGAACTAATGAAAAATGTTAGCCACAATCCCCTTTTACTTCTCGCACCACAGAAAGTTAAAAGATATGTTGAGTCTTTATGGAAGAAGAAAAGCTCTGGACAACCTGTCACCTTTACTGATATC) oligo containing Ala-268 was ligated into the EcoRI-EcoRV site to create pcDNA3.1-CPLA2A4T. pcDNA3.1-CPLA2 was digested with BmgB1 plus PvuII, and a 116 bp linker-oligo (GACGTGCTGGGAAGGTACACAACTTCATGCTGGGCTTGAATCTCAATACATCTTATCCACTGGCACCTTTGAGTGACTTTGCCACACAGGACTCCTTTGATGATGATGAACTGGATGCAGCTG) containing Ala-505 was ligated into the BmgB1 plus PvuII site to create pcDNA3.1-CPLA2A4S. PCR was performed on pcDNA3.1-CPLA2, pcDNA3.1-CPLA2 A4T, and pcDNA3.1-CPLA2 A4S to amplify the cPLA2 genes, which were ligated into pFB6XHis (made in this laboratory) to create pFB6XHIS-CPLA2A4T, pFB6XHIS-CPLA2A4T, and pFB6XHIS-CPLA2A4S. These plasmids contain hcPLA2 wt, Ala-268, and Ala-505, respectively, preceded by 6 histidine residues, which allow for affinity chromatography purification of the proteins. The Ala-505 sequence was removed from pFB6XHIS-CPLA2A4S by restriction enzyme digestion and was used to replace the Ser-505 sequence in pFB6XHIS-CPLA2A4T to create the double mutant, Ala-268 and Ala-505, pFB6XHIS-CPLA2DM. The 6XHIS-CPLA2 sequences were PCR amplified out of pFB6XHIS constructs and ligated into plasmodium dihydrofolate reductase (pDHFR; catalog #01938, New England BioLabs) after removal of the *Escherichia coli* DHFR gene to create pDHFR-CPLA2, pDHFR-CPLA2A4T, pDHFR-CPLA2A4S, and pDHFR-CPLA2DM. The sequences of all constructs were confirmed by plasmid DNA sequencing.

### *In vitro* protein expression and purification

The pDHFR-cPLA2 wt, cPLA2A4T (cPLA2 T268A), cPLA2A4S (cPLA2 S505A), and cPLA2 DM plasmids were used for *in vitro* transcription/translation using the PURExpress *in vitro* Protein Synthesis Kit (catalog #E6800S, New England BioLabs). Proteins (wild-type, two single mutants and double mutants) were purified using 50 kDa an Amicon Ultra-0.5 Centrifugal Filter Unit (Millipore). The products were confirmed using SDS-PAGE both before and after purification.

### *In vitro* protein expression (mammalian cells)

The CMV promoter was inserted upstream of the cPLA2 sequences in the pDHFR constructs, which allows the plasmids to express the cPLA2 proteins in mammalian cells.

### Primary cortical astrocytes and mesencephalic cell culture and treatment

Cultures were prepared from the cortical tissues of embryonic day 18.5, as described previously by Schildge et al. (2013). Twelve to 14 d after the first split, astrocytes were pretreated with or without TFP5 (a Cdk5 inhibiting peptide with amino acid sequence FITCGGGKEAFWDR-CLSVINLMSSKMLQINAYARAARRAARR) (500 nm) or scrambled peptide (SCP) for 12 h, then coincubated in a 10 μm/ml concentration of MPP^+^ and with or without TFP5 (500 nm) or SCP for 24 h. The mesencephalic neuron–glia cultures were prepared from C57BL6/J mice using a method reported by Binukumar et al. (2012). The MPP^+^ and TFP5 treatment are also the same as reported previously.

### Secondary cell culture

Human embryonic kidney 293T (HEK293T) cells were cultured in DMEM media (catalog #12100046, Thermo Fisher Scientific), with 10% fetal bovine serum (catalog #10437028, Thermo Fisher Scientific) and 1× penicillin/streptomycin (catalog #15140122, Thermo Fisher Scientific). The cells were tested negative for mycoplasma using luminescence-based mycoplasma detection kit (catalog #LT07-418, Lonza).

### Expression of cPLA2 mutants

The cPLA2 constructs along with Cdk5/p35 and Cdk5/p25 were cotransfected using lipofectamine-3000 in HEK293T cells. A total of 1.5 μg of plasmid was added along with the 1× the volume of P3000 and 3× the volume of lipofectamine-3000 per well in serum-free media (catalog #L3000015, Thermo Fisher Scientific). The cells were harvested 24 h post-transfection using RIPA buffer and 1× protease inhibitor cocktail (protease inhibitor cocktail tablets, Sigma-Aldrich). The concentration of the proteins was estimated through BCA protein estimation assay.

### Generation of phospho-cPLA2(pcPLA2T268) antibody

The custom-made pcPLA2T268 antibody was generated by the GenScript Antibody Group [the cPLA2_268-phospho-antigen 1-(LLL{pTHR}PQKVKRYVESC); lot 779114-1, GenScript; the cPLA2_268- non-phospho-antigen 2 (LLLTPQKVKRYVESC), lot: 779115-1, GenScript; Peptide (L>OT: 779114-1)-KLH conjugate, ImmunoGen; host stain, New Zealand Rabbit].

The cPLA2_268-phospho-antigen 1-(LLL{pTHR}PQKVKRYVESC) with keyhole limpet hemocyanin (KLH) conjugate was injected into the New Zealand rabbit for immunization. The serum was isolated and subjected to cPLA2 non-phosphopeptide affinity column for subtraction. The flow through was further subjected to the phosphopeptide affinity column for affinity purification. The antibodies binding to the phosphopeptide column was eluted and characterized further through indirect ELISA. For antibody characterization, the phosphopeptides and nonphosphopeptides were coated with 4 μg/ml, 100 μl/well concentration with PBS, pH 7.4, as coating buffer. The secondary antibody used in the ELISA was anti-rabbit IgG monoclonal antibody (HRP conjugate; catalog #AO1827, GeneScript).

### Western blot analysis

The HEK293T cell protein lysate as well as brain lysates from control (*n *=* *6) and PD (*n *=* *6) mice were prepared as described previously (Shukla et al., 2017). Polyacrylamide gel running, nitrocellulose membrane transfer, and detection were performed as reported previously (Shukla et al., 2017). The antibodies used in the Western blot experiment are 6×-His tag (1 μg/ml; catalog #NBP2-61482, Novus Biologicals) for cPLA2 variants, Calnexin (1:5000; stock dilution, 1 mg/ml; catalog #SAB4503258, Sigma-Aldrich), cPLA2 (1:1000; stock dilution, 1.5 mg/ml; catalog #SAB4200211, Sigma-Aldrich), p-cPLA2-Ser-505 (1:1000; stock dilution, 1 mg/ml; catalog #SAB4503812, Sigma-Aldrich), Cdk5 (1:1000; stock dilution, 5 μg/ml; catalog #AHZ0492, Thermo Fisher Scientific), p35/25 (1:5000; stock dilution, 1 mg/ml; catalog #C64B10, Cell Signaling Technology), p-cPLA2-Thr-268 (1:500; GenScript Biotech), and β-tubulin (1:10,000; stock dilution, 1 mg/ml; catalog #T8328, Sigma-Aldrich). The blots were developed using chemiluminescence detection through an ECL substrate (catalog #32109, Thermo Fisher Scientific). The blots that probed both Ser505 and Thr268 with phospho-cPLA2 were reprobed with total cPLA2 antibodies. All the other blots used were cut according to their molecular weight with the help of a ladder and probed with different antibodies. The intensity of the blots was analyzed using an ImageJ tool.

### Immunoprecipitation of cPLA2 proteins

The protein lysate post-transfection in HEK293T cells was subjected to immunoprecipitation using A/G Sepharose beads (catalog #786–836, G-Biosciences). The beads were bound to an anti-6×-His tag antibody (2 μg/μl) overnight at 4°C and then washed three times using a wash buffer (50 mm Tris, pH 8.5, 1 mm EGTA, 75 mm KCl). The 6×-His tag bound beads were then incubated with the 170 μg of protein lysate (HEK293T and PD brain protein lysate) for 5 h at room temperature. The beads along with their bound antigen were collected by centrifugation at 13,000 rpm at 4°C for 20 min, and the supernatant was discarded. The collected beads were washed again three times with the wash buffer. The product was eluted using urea elution buffer (7 m urea; 20 mm, Tris pH 7.5; and 100 mm NaCl).

### cPLA2 activity assay

The cPLA2 assay of the mutants expressed in HEK293T cells along with the cPLA2 expressed in PD brain was performed using a cPLA2 assay kit (catalog #ab133090, Abcam). Fifteen microliters (30 μg) of protein lysate samples was added in 96-well plates. The reaction was initiated using 200 μl of Arachidonoyl Thio-PC (substrate solution) and incubated for 60 min. Ten microliters of DTNB/EGTA was added to each well to stop enzyme catalysis and develop the reaction. The absorbance was measured at 414 nm, and the cPLA2 activity was calculated as described in the kit protocol. All the reactions were performed in triplicate.

### Cdk5 kinase assay

The Cdk5 kinase assay was performed with the immunoprecipitated product with Cdk5 antibody in the HEK293T cells as well as the brain protein lysate of PD mouse model. The assay was based on the work in previously published articles (Binukumar et al., 2014, 2015; Shukla et al., 2017) and by using the ADP-GloTM Kinase Assay Kit (catalog #V6930, Promega).

### Cytosolic and membrane protein fractionation

The HEK293T cells were harvested 24 h post-transfection. The cytosolic and membrane protein fractionation was done using the Mem-PER Plus Membrane Protein Extraction Kit (catalog #89842, Thermo Fisher Scientific). The cells from each well were collected using a scraper followed by centrifugation at 300 × *g* for 5 min. The cell pellets were each washed with 200 μl of wash buffer twice. The cell pellets were then treated with a 100 μl/well permeabilization buffer for 10 min at 4°C with constant mixing. The permeabilized cells were centrifuged at 16,000 × *g* for 15 min at 4°C. The supernatants containing the cytosolic fraction were collected. The pellet was further treated with 100 μl of solubilization buffer and incubated for 30 min at 4°C with constant mixing. The solubilized membrane fraction was then centrifuged at 16,000 × *g* for 15 min at 4°C. The supernatant containing solubilized membrane-associated protein was collected.

### Prostaglandin E2 ELISA

The prostaglandin E2 (PGE2) ELISA was performed using the Prostaglandin E2 ELISA Kit (catalog #KHL1701, Thermo Fisher Scientific). The reaction mixture for the sample was made in PGE2-coated eight-well strips where 60 μg of brain protein lysate samples was added along with 50 μl of PGE2-AP tracer and 50 μl of PG2 monoclonal antibody supplied within the kit. Along with the sample reaction, a standard reaction was also set up for PGE2 with dilutions as required. After 2 h of incubation, 200 μl of pNPP (4-nitrophenyl phosphate) solution was added, and again an incubation of 70 min was performed in complete darkness. Absorbance at 405 nm was measured, and the PGE2 (in picograms per milliliter) was quantified in the sample using the calculations given in the protocol.

### Immunocytochemistry

HEK293T cells were seeded in poly-d-lysine-coated glass coverslips placed in a 12-well plate. After 12 h of seeding, the cells were cotransfected with cPLA2 variants along with Cdk5/p35 and Cdk5/p25, respectively. Twenty-four hours post-transfection, the cells were fixed with chilled 100% methanol for 5 min. After fixation, the cells were incubated with a blocking solution (1% BSA, 22.52 mg/ml glycine in PBST) for 30 min. The cells were then incubated with cocktail primary antibodies cPLA2 (1:250; stock dilution, 1.5 mg/ml; catalog #SAB4200211, Sigma-Aldrich) and Cdk5 (1:250; stock dilution, 5 μg/ml; catalog #AHZ0492, Thermo Fisher Scientific) for 1 h in a humid chamber. After incubation with primary antibodies, the cells were washed with PBS three times and incubated with secondary anti-mouse 488 nm (0.2 μg/ml; catalog #A-10667, Thermo Fisher Scientific) and anti-rabbit 594 nm (0.2 μg/ml; catalog #A32740, Thermo Fisher Scientific) for 1 h. The cells were then washed with PBS three times, mounted with DAPI (1 μg/ml; catalog #D9542, Sigma Aldrich), and sealed in glass slides. All the images were captured using an inverted microscope (model Ti-E, Nikon).

### Immunohistochemistry protocol

Ten micrometer cryostat sections of the brain were collected on slides and prepared for immunohistochemistry, which was performed according to standard protocols for single or double immunostaining (Binukumar et al., 2015). The following primary antibodies were used: Pcpla2 S505 (1:100; stock dilution, 1 mg/ml; catalog #SAB4503812, Sigma-Aldrich); Pcpla2 T268 (1:50); cPLA2 (1:250; stock dilution, 1.5 mg/ml; catalog #SAB4200211, Sigma-Aldrich); and GFAP (1:300; stock dilution, 1 mg/ml; catalog #PA1-10004, Thermo Fisher Scientific). Immunostaining was visualized by TRITC (549 nm; 1:500; anti-mouse) and FITC (488 nm; 1:500; anti-rabbit) Alexa Fluor secondary antibodies (Thermo Fisher Scientific) and was examined using inverted microscopy (model Ti-E microscope, Nikon).

### PD patients transcriptome data analysis

The conjoint analysis of the PD Patients was conducted to check the expression profiles of the arachidonic acid, prostaglandin synthesis pathway, and inflammatory pathway genes in the context of humans. We used “Parkinson’s disease” as the search term to screen for the desired datasets on NCBI (National Center for Biotechnology Information)-GEO (Gene Expression Omnibus) and the ArrayExpress database. The datasets were chosen such that they belonged to the substantia nigra of the PD patients. The following datasets were used in the analysis: GSE8397 (Moran et al., 2006), GSE20141 (Zheng et al., 2010), GSE7621 (Lesnick et al., 2007), and GSE20163 (Lesnick et al., 2007; Zheng et al., 2010). Before differential expression analysis, we have performed preprocessing, log2 transformation and normalization according to the downloaded expression matrix. Preprocessing involved (1) common probes expression profiles and (2) rescaling of the data; 21,941 common probes corresponding to 13,193 genes were used for rescaling expression profiles to 1 (minimum value). Then we performed log_2_ transformation on the profiles. Normalization of the data was performed using surrogate variable analysis. We used the ComBat function of the sva package (Leek et al., 2012) to remove the batch-specific or study-specific effects and retain biological effects (expression profile related to PD and healthy control mice). To inspect the normalization of the expression profiles, we performed principal component analysis on the normalized expression profiles and on those profiles without normalization expression profiles (see Fig. 10). The normalized expression profile was further used for differential expression using the eBayes (Smyth, 2004; Johnson et al., 2007) method available in the limma (Ritchie et al., 2015) package in R.

### Statistical analysis

All datasets underwent statistical analysis with GraphPad Prism 8. Every dataset was displayed as mean ± SEM. Unpaired *t* tests with the Holm–Sidak method and a one-way ANOVA followed by Bonferroni’s *post hoc* test were used to demonstrate the statistical significance between the groups of animals. The individual figure legends describe the test performed for each analysis. The figure legends have highlighted statistical trends (*p* values that are significant; *p = 0.05, **p = 0.01, and ***p = 0.001) between the indicated groups.

## Results

### Cdk5 phosphorylates at S-505 and T-268 of cPLA2

**Figure 1.**
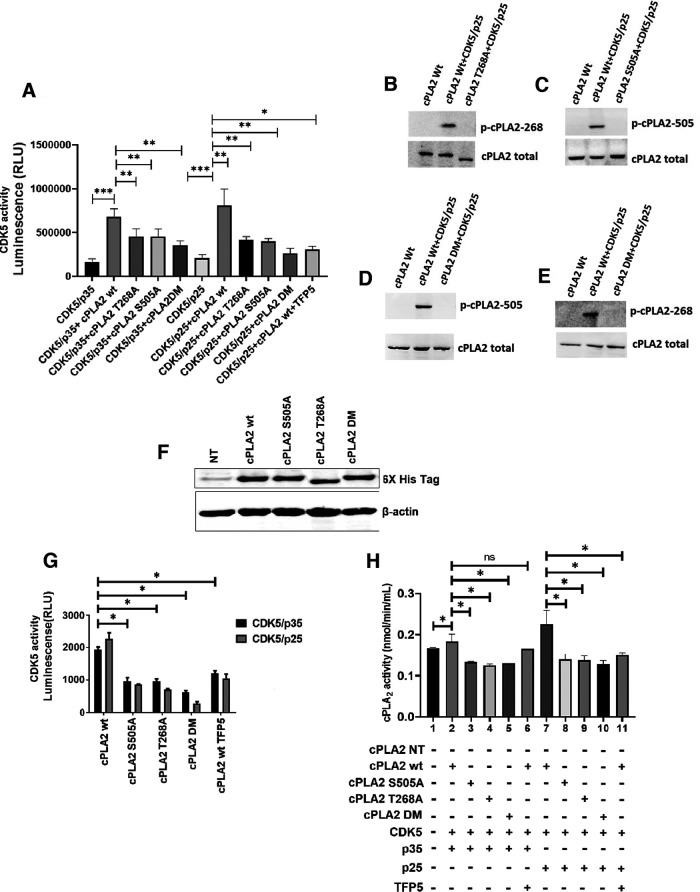
cPLA2 phosphorylation by Cdk5. ***A***, Cdk5 kinase assay with cPLA2 wt and its mutants along with TFP5 treatment *in vitro* by its pure proteins. ***B–E***, Western blot images of phosphorylated cPLA2 (i.e., p-cPLA2 268 and p-cPLA2 505 expressions after Cdk5 kinase assay with cPLA2 wt and its mutants as substrate). Total cPLA2 was considered as standard. ***F***, The Western blot image shows the expression of cPLA2 and its mutants post-transfection in HEK293T cells. ***G***, Bar diagram showing Cdk5 kinase activity with cPLA2 wt and mutant protein as substrates of Cdk5 along with Cdk5 inhibitor TFP5. ***H***, cPLA2 kinase activity. The bar graph shows cPLA2 activity of cPLA2 wt along with its three mutants S505A, T268A, and DM, also a comparative analysis of its mutants with the cPLA2 wt activity. Data are presented as the mean ± Standard error of mean (SEM), and analysis was performed using one-way ANOVA and Bonferroni’s test; *n* = 3. *p < 0.05, **p < 0.01, ***p < 0.001, not significant (ns).

To show whether cPLA2 is a substrate for Cdk5 phosphorylation at Ser-505 and Thr-268, the single mutant and double mutant plasmids were generated. Ser-505 and Thr-268 are replaced by alanine (A) creating cPLA2 single mutants S505A, T268A, and double mutant (S505A, T268A). These plasmids were used for *in vitro* transcription/translation for pure protein synthesis. The pure proteins were used in the Cdk5 kinase assay. The kinase assay data (Fig. 1*A*) indicate that Cdk5/p25 is significantly more effective in phosphorylating cPLA2 wt than Cdk5/p35. It must be highlighted that both the single mutants, S505A and T268A, show decreased phosphorylation when compared with cPLA2 wt. While a double mutant of cPLA2 shows decreased phosphorylation compared with the corresponding single mutations. Moreover, the cPLA2 wt phosphorylation is halted in the presence of a Cdk5 inhibitor. This assay uncovers that S-505 and T-268 are the potential Cdk5 phosphorylation sites on cPLA2 (Fig. 1*A*). Again, to check the presence of cPLA2 phosphorylation at Ser-505 and Thr-268 after kinase activity with Cdk5/p25, a Western blot was performed using phospho-specific antibodies p-cPLA2-505 and p-cPLA2-268. The result supports the phosphorylation of cPLA2 wild-type both in S-505 and T-268 sites in the presence of Cdk5 and p25. Also, p-cPLA2-505 and p-cPLA2-268 are absent in the single and double mutant cPLA2 when used as substrates with Cdk5 and p25 (Fig. 1*B–E*).

To authenticate the above *in vitro* findings, cPLA2 phosphorylation by Cdk5, the expression of the cPLA2 wt, as well as mutants, is confirmed by its transfection in HEK293T cells followed by Western blot using a 6×-His tag antibody (Fig. 1*F*). The overexpressed cPLA2 wt and mutant proteins were pulled down using immunoprecipitation with 6×-His tag antibody and subjected to Cdk5 kinase assay. The *in vitro* Cdk5 kinase assay results (Fig. 1*G*) also show decreased phosphorylation in all the cPLA2 mutants. Previously, it has been reported that S-505 was a phosphorylating site of MAPK. Here, we are first reporting that S-505 and T-268 are the novel Cdk5 phosphorylation sites on cPLA2.

### S-505 and T-268 of cPLA2 are critical for its activity

Subsequently, we ensure whether these sites are vital for cPLA2 kinase activity. We overexpressed all the cPLA2 plasmids with Cdk5/p35 and Cdk5/p25 along with the TFP5/SCP treatment, and pulled down the cPLA2 proteins with a 6×-His tag for the cPLA2 kinase assay through immunoprecipitation (Fig. 1*G*). The result shows a decrease in cPLA2 activity in two single mutants along with the double mutant compared with the cPLA2 wt (Fig. 1*H*). This indicates that both sites, S-505 and T-268, are important for the phospholipase activity of cPLA2; therefore, mutation in any of the sites or both the sites together affects the cPLA2 activity significantly. Moreover, phosphorylation of these sites enhances the cPLA2 activity (Fig. 1*H*). The Cdk5 kinase-specific inhibitor treatment shows decreased cPLA2 wt kinase activity.

### Comparison of wild-type and mutant structural cPLA2 using MD simulations

To obtain a deeper understanding of how mutations might affect the functional state of the protein, we performed microsecond-scale molecular dynamics simulations of cPLA2 enzyme. Structurally, the two phosphorylated sites are on different faces of protein, with amino acid position S-505 in the cap region of cPLA2, which is known to be highly flexible (Dessen et al., 1999), and Thr-268 at the nucleophilic elbow. In parallel, we also generated three mutant simulations, namely T268A, S505A, and a double mutant with both positions substituted to an alanine residue. The mutant simulations showed an increase in the angle between two functional domains of ∼100°, a significant increase from 120° observed in the wild-type structures. While the nonmutant structures are almost of open configuration (large angular shift), domains in mutant structures are drastically different and represent a more “compact” state.

### Secondary structure evolution of the catalytic domain residues (144–749) according to DSSP analysis in wild and mutant trajectories

**Figure 2.**
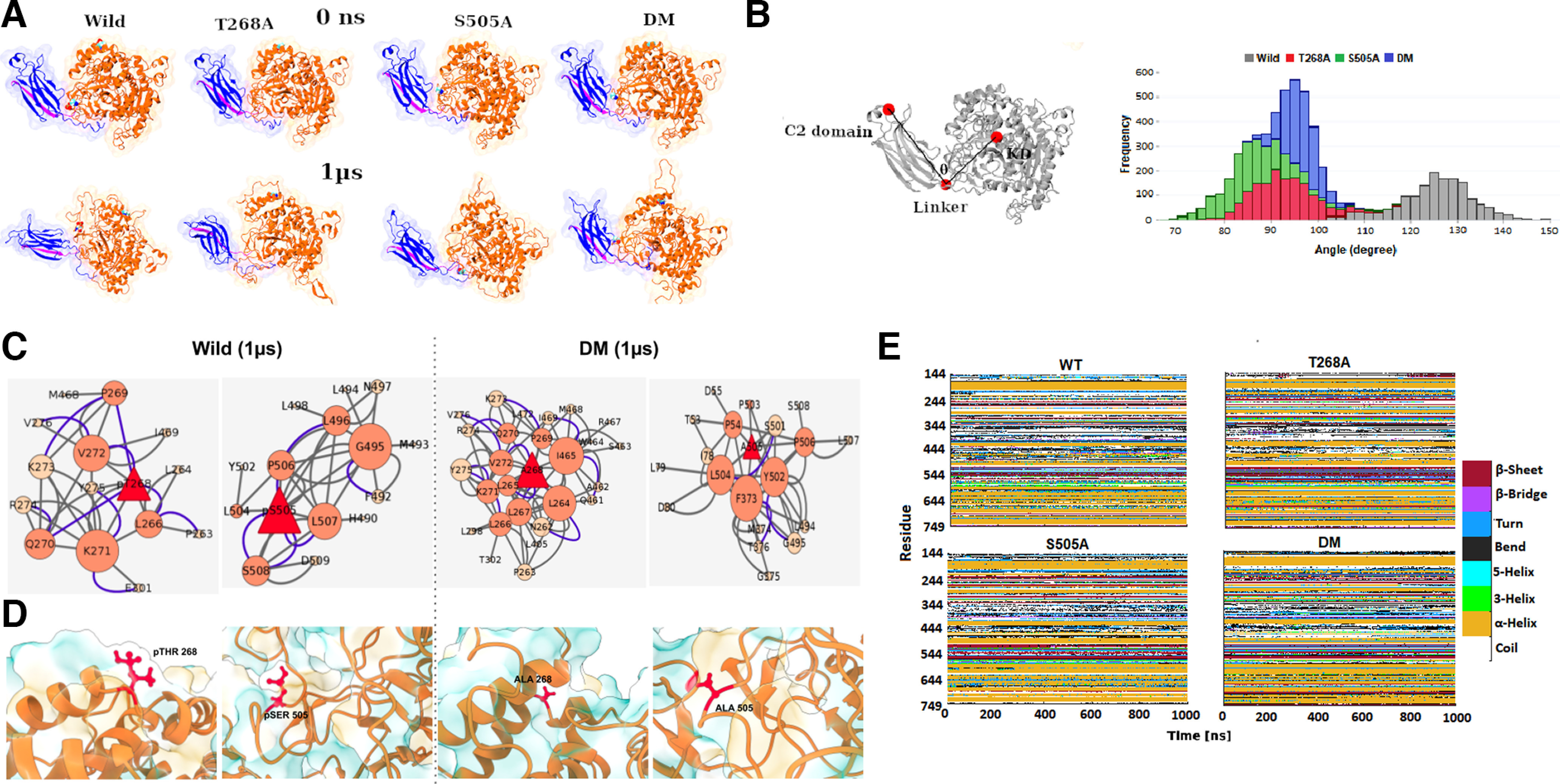
Comparison of wild-type and mutant structural forms using MD simulations. ***A***, Representative snapshots of MD simulations derived structure at the initial (0 ns) and last (1 μs) of wild and mutant systems. The protein structures are colored according to cPLA2 domain architecture, where C2 domain is highlighted in blue, linker in magenta, and catalytic domain (KD) in orange. ***B***, Angular distribution between domains in wild and mutant systems during the last 150 ns of the trajectory. ***C***, Secondary structure evolution of the catalytic domain residues (144–49) according to DSSP analysis in wild and mutant trajectories. ***D***, Intramolecular network near the mutant sites for wild and double mutant systems at 1 μs. The size of the nodes represents the degree. The edges are represented as types of interaction, where hydrogen bonds are highlighted in blue, and contacts in gray. ***E***, Snapshots highlighting these sites in ball and stick representation and surface hydrophobicity shown in blue and gold.

To further understand local differences, we calculated secondary structural elements and obtained noticeable changes at the active site region (Fig. 2*C*). For instance, the residues between 410 and 430 occur as coils in wild type, which form short helical structures in mutant trajectories. These findings suggested a shift in the contacts because of these critical amino acid substitutions. Further, we analyzed the network of residue interaction at each of the mutant sites by quantifying the number of contacts and hydrogen bonds. We observed the number of overall interactions increased for the double mutant system compared with the wild type. The network topology derived from the structures at 1 μs suggests that the networks are dense with additional first neighboring nodes around the central mutant site in case of a double mutant. For instance, residue 268 has more neighboring nodes in the double mutant compared with the wild type. On a closer look, we found that these result in backbone changes. Rendering the phosphorylated sites according to surface accessibility revealed that the phosphorylated residues that are exposed in the wild-type protein are inaccessible in the double mutant protein (Fig. 2*A*,*B*). Comparison of the phosphorylated Ser-505 residue, which is a major hub in wild type, suggested a loss of interaction with 12 interactions in wild-type, whereas only 6 interactions were observed in the double mutant. Our network analysis suggests that mutation of these phosphorylating residues results in dynamic changes around the mutant sites, which may affect the activity of the protein (Fig. 2*D*,*E*).

### Membrane trafficking of cPLA2 is hindered in its mutants T268A and S505A

**Figure 3.**
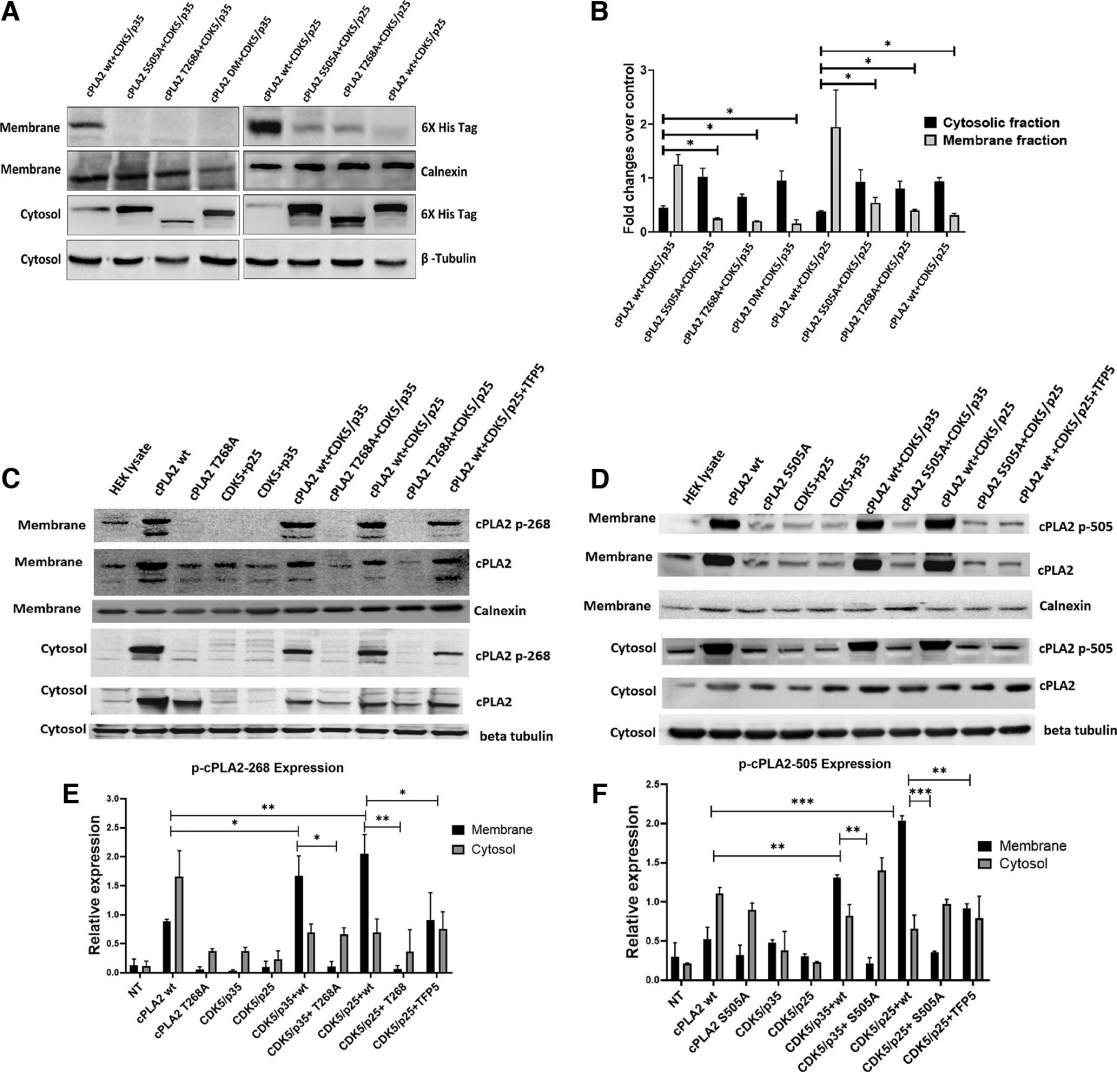
The expression of cPLA2 wt and its mutants in the membrane and cytosolic protein fraction. ***A***, The expression pattern of cPLA2 and its mutants in a membrane and cytosolic fraction of HEK293T protein lysate cotransfected with cPLA2 and its mutants along with Cdk5/p35 and Cdk5/p25. The reaction standard used for membrane fraction is calnexin, and for cytosolic fraction is β-tubulin. ***B***, Cytosolic and membrane fraction fold changes over control. ***C***, Membrane translocation of phosS268 cPLA2 in the presence of active Cdk5 kinase. ***D***, Membrane translocation of phospho-T505 cPLA2 in the presence of active Cdk5 Kinase. ***E***, Densitometry of relative expressions of p-cPLA2-268 in cytosolic and membrane fractions. ***F***, Densitometry of p-cPLA2-505 in cytosolic and membrane fractions. Calnexin is used as membrane fraction control, and β-tubulin as cytosolic frantic control. Data are presented as the mean ± SEM, and analysis was performed using a one-way ANOVA followed by Bonferroni’s *post hoc* test; *n* = 3. *p < 0.05, **p < 0.01, ***p < 0.001.

Next, we hypothesize phosphorylation on these sites is responsible for the plasma membrane translocation of cPLA2. The hypothesis predicts that cPLA2 is activated on phosphorylation by Cdk5. The membrane and cytosolic protein fractions are collected post-transfection, and the expression pattern of cPLA2 wt and mutant mice are assessed (Fig. 3). Using the His tag antibody for Western analysis of each fraction, the result shows that the amount of cPLA2 wt in the membrane fraction is more comparable to cPLA2 T268A, S505A, and DM, which are predominantly retained in the cytosolic fraction (Fig. 3*A*,*B*). Therefore, cPLA2 phosphorylation at T-268 and S-505 by Cdk5 is critical for its translocation from cytosol to the plasma membrane. An antibody for phospho-cPLA2-S-505 is available commercially; however, the phospho-cPLA2 T-268 antibody was custom synthesized. Figure 3*A* shows phospho-T-268 and phospho-S-505 cPLA2 move to the plasma membrane in the presence of Cdk5/p35 and Cdk5/p25 at the same time the mutant fails to translocate to the plasma membrane. This is the key experiment that demonstrates that cPLA2 wt is indeed phosphorylated by Cdk5 and phosphorylated cPLA2 at T-268 is translocated to the membrane fraction, but the mutant cPLA2 T268A is absent in the membrane fraction. In addition, pretreatment with Cdk5 inhibitor TFP5 in cells potentiates the effect of Cdk5 on the level of plasma membrane-associated cPLA2 wt (Fig. 3*C*,*E*). The increase in the amount of cPLA2 wt associated with the plasma membrane correlates well with the decrease in the amount of cPLA2 wt found in the cytosol following Cdk5/p25 and Cdk5/35 overexpression, therefore suggesting the translocation of phosphorylated cPLA2 wt from the cytosol to the plasma membrane on activation. In the case of cPLA2 S-505 phosphorylation, we also get similar results (Fig. 3*D*,*F*).

### cPLA2 and Cdk5 colocalization and interaction

**Figure 4.**
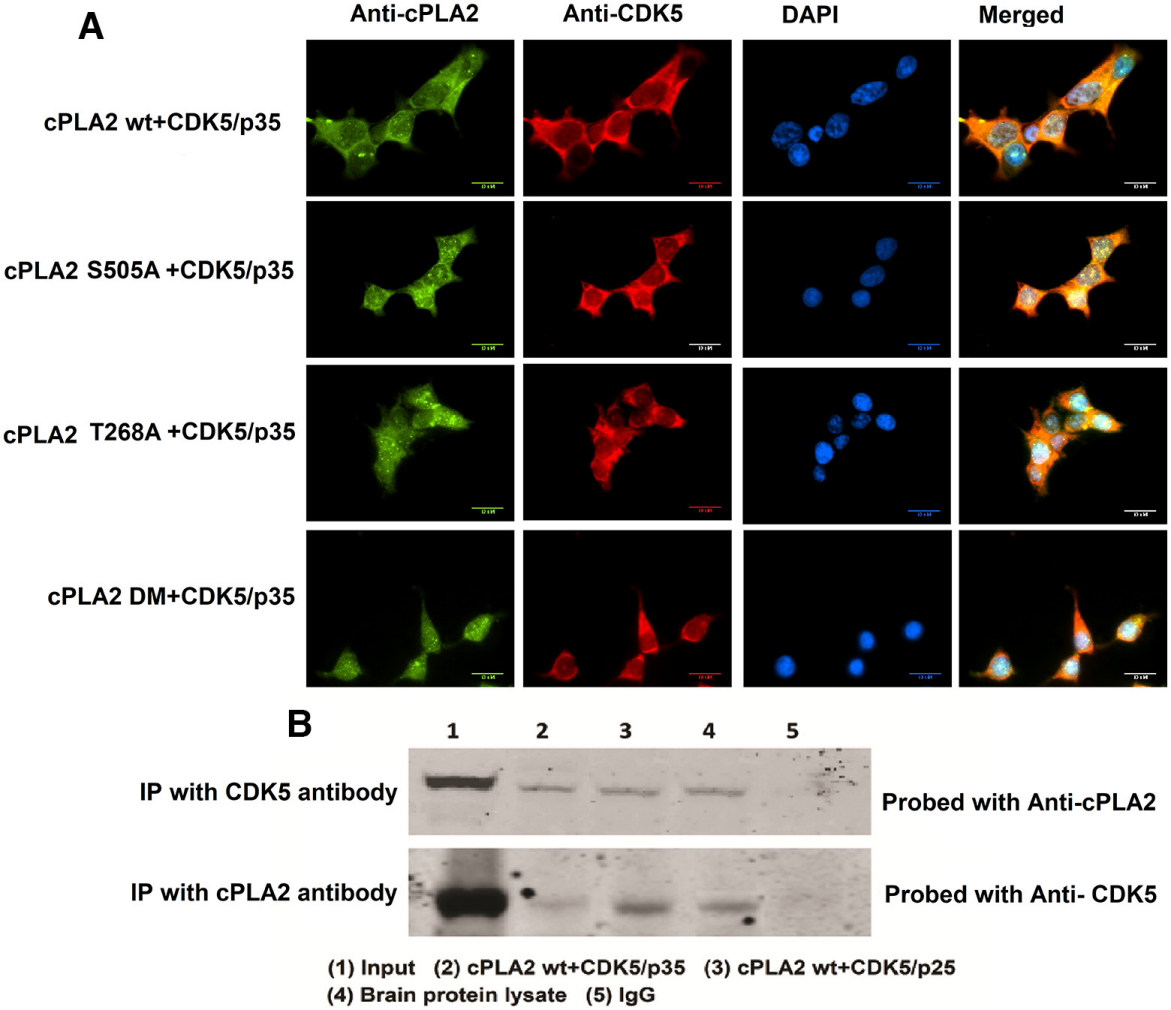
Colocalization of Cdk5 and cPLA2. ***A***, Colocalization of Cdk5 and cPLA2. Immunocytochemistry analysis of Cdk5 and cPLA2 showing their expressions and colocalization. ***B***, Western blot data of Cdk5 and cPLA2 pull-down IPs developed with cPLA2 and Cdk5 antibodies, respectively.

The immunocytochemistry data show the expression of the cPLA2 wt and mutants in the presence of Cdk5/p35 and Cdk5/p25 (Fig. 4*A*). The mutants were present in the cytosol even in the presence of active Cdk5 kinase. Also, Cdk5 is shown to colocalize with cPLA2. Moreover, coimmunoprecipitation experiments were conducted with mouse brain lysates and Cdk5, and with cPLA2 overexpressed in the HEK293T cell lysate. Western blotting with Cdk5 immunoprecipitation products (IPs) and cPLA2 IPs using antibodies for cPLA2 and Cdk5, respectively, shows that Cdk5 physically interacts with cPLA2 (Fig. 4*B*).

### Enhanced cPLA2 activity in primary cortical astrocytes culture exposed to MPP^+^ via Cdk5

**Figure 5.**
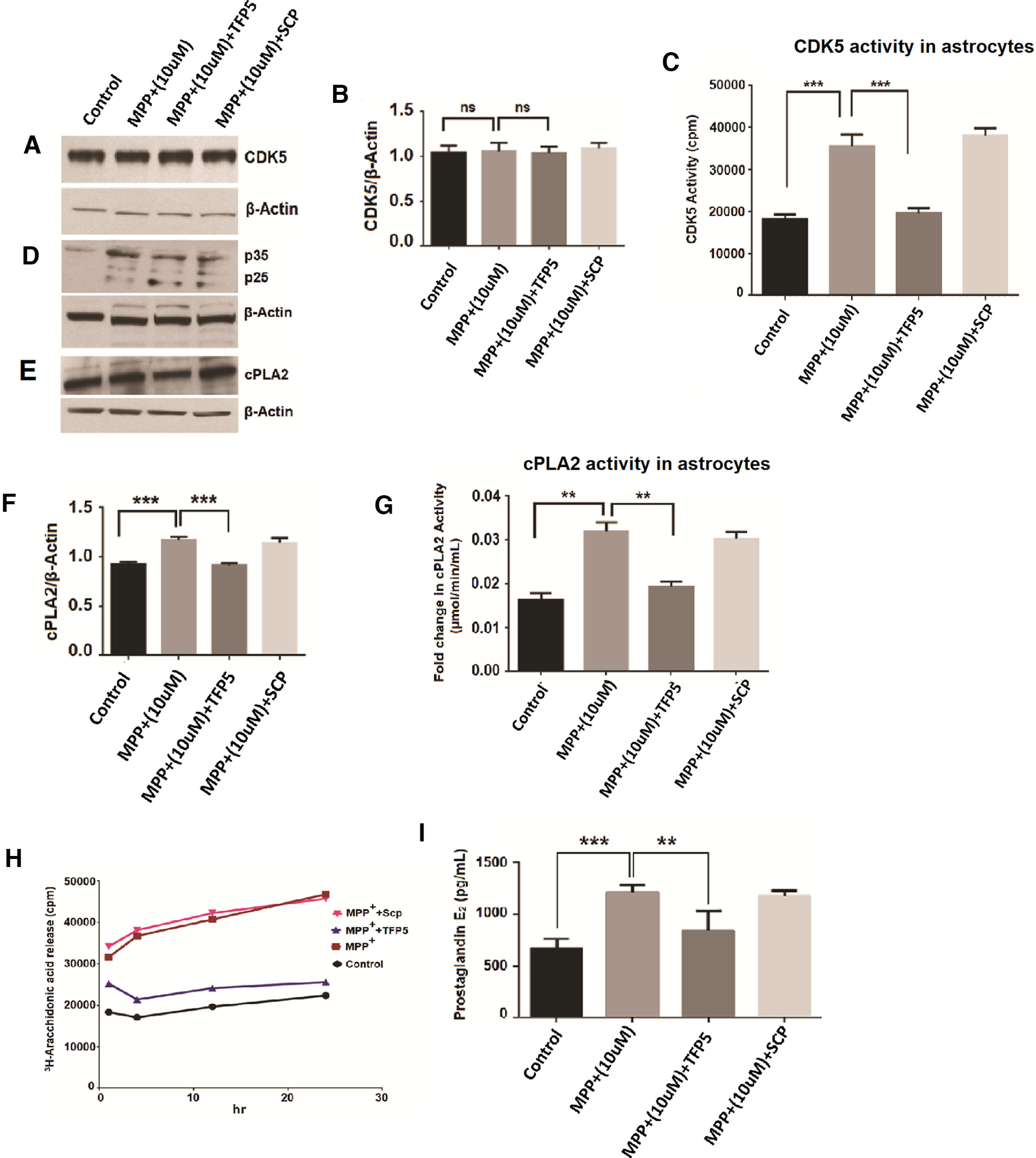
Cdk5 expression, generation of p25, its kinase activity, and release of arachidonic acid and prostaglandin E2 synthesis by astrocytes exposed to MPP^+^ and TFP5 treatment. ***A***, The image shows a blot with the expression of Cdk5 in control versus MPP^+^ (10 μm) along with MPP^+^ (10 μm) versus MPP^+^ (10 μm) plus TFP5 and SCP treatment in the protein lysate of astrocytes. ***B***, Bar diagram showing densitometric analysis of Cdk5 expression over β-actin used as a standard for control, MPP^+^ (10 μm), MPP^+^ (10 μm) + TFP5 and MPP^+^ (10 μm) + SCP, respectively. ***C***, The bar diagram represents Cdk5 activity in the astrocyte protein lysate with control versus MPP^+^ (10 μm) and MPP^+^ (10 μm) versus MPP^+^ (10 μm) + TFP5. ***D***, The expression pattern of p35 and p25 in control, MPP^+^ (10 μm), MPP^+^ (10 μm) + TFP5, and MPP^+^ (10 μm) + SCP, respectively. β-Actin was used as a standard. ***E***, The blot representing the expression pattern of cPLA2 in control, MPP^+^ (10 μm), MPP^+^ (10 μm) + TFP5, and MPP^+^ (10 μm) + SCP, respectively. ***F***, The densitometric graph showing cPLA2 expression over β-actin of the above blot. ***G***, The bar graph showing fold changes in cPLA2 activity in astrocyte lysate of control versus MPP^+^ (10 μm) and MPP^+^ (10 μm) versus MPP^+^ (10 μm) + TFP5. ***H***, The scatter plot showing 3H-arachidonic acid release from control astrocytes along with MPP^+^ (10 μm), MPP^+^ (10 μm) + TFP5, and MPP^+^ (10 μm) + SCP. ***I***, The bar graph showing prostaglandin E2 synthesis in control versus MPP^+^ (10 μm) and MPP^+^ (10 μm) versus MPP^+^ (10 μm) + TFP5 along with MPP^+^ (10 μm) + SCP as the standard for inhibitor treatment. Data are presented as the mean ± SEM, and analysis was performed using one-way ANOVA and Bonferroni’s test; *n* = 3. *p < 0.05, **p < 0.01, ***p < 0.001, not significant (ns).

Previous studies showed high expression of Cdk5 in astrocytes in addition to its main role in neurons. We also previously reported that MPP^+^ exposure to midbrain culture leads to the deregulation of Cdk5/p25 activity (Binukumar et al., 2014). The primary astrocytes were cultured (Schildge et al., 2013) and pretreated with Cdk5 inhibitor TFP5 (500 nm) or SCP for 12 h. Then coincubated with 10 μm MPP^+^ for 24 h. We could show that Cdk5 is expressed in these cells and the level of expression is unaffected by neither the treatment nor the presence of TFP5 (Fig. 5*A*,*B*). Cdk5 activity, however, was enhanced in the presence of MPP^+^ and reduced in the presence of TFP5 peptide (Fig. 5*C*). Likewise, p25 generation (Fig. 5*D*) was correlated with increased Cdk5 activity (Fig. 5*C*), which was restored to control values after TFP5 treatment. cPLA2 activity was measured after stimulation of primary astrocytes with MPP^+^ and Cdk5 inhibitor pretreatment. We confirmed that cPLA2 was expressed in astrocytes (Fig. 5*E*,*F*) and MPP^+^ treatment leads to the twofold activation of cPLA2 activity compared with untreated cells (Fig. 5*G*). The observed stimulated cPLA2 activity is inhibited by Cdk5 inhibitor; however, SCP pretreatment has no effect on the stimulated activity of cPLA2 (Fig. 5*G*).

### Active cPLA2 induces arachidonic acid release and enhanced prostaglandin E2 synthesis: initiation of an inflammation cascade is inhibited by Cdk5 inhibitor pretreatment

To investigate whether activated cPLA2 releases high levels of AA into the culture medium and whether TFP5 treatment could reduce its release, we prepared astrocyte cultures from rat brains and quantified AA. Media from cultured cells was collected at different time points (30 min, 1 h, 4 h, 12 h, and 24 h) and assayed in a scintillation counter. MPP^+^-treated cells showed increased AA release compared with controls. TFP5 treatment showed significantly less AA radioactivity in the medium compared with the MPP^+^ treatment group (Fig. 5*H*). Figure 4, *H* and *I*, summarizes the results on the role of potential Cdk5-mediated cPLA2 activation and TFP5 in ameliorating factors contributing to neuroinflammation in astrocytes because of MPP^+^ treatment.

To explore PGE2 synthesis in MPP^+^ treated cells, the embryonic rat astrocytes were cultured for 12–14 d. The cells were pretreated with/without TFP5 (500 nm) or SCP for 12 h followed by coincubation in 10 μm MPP^+^, with or without TFP5 (500 nm) or SCP for 24 h. Prostaglandin synthesis was quantified from the supernatant. MPP^+^-treated cells showed increased levels of prostaglandin E2 compared with controls. Cdk5 inhibitor treatment ameliorates this effect and determines whether SCP treatment has any effect on the prostaglandin E2 level (Fig. 5*I*).

### Increased cPLA2 activity and enhanced prostaglandin production in neuronal–glial cultures exposed to MPP^+^ associated with deregulated Cdk5 activity

**Figure 6.**
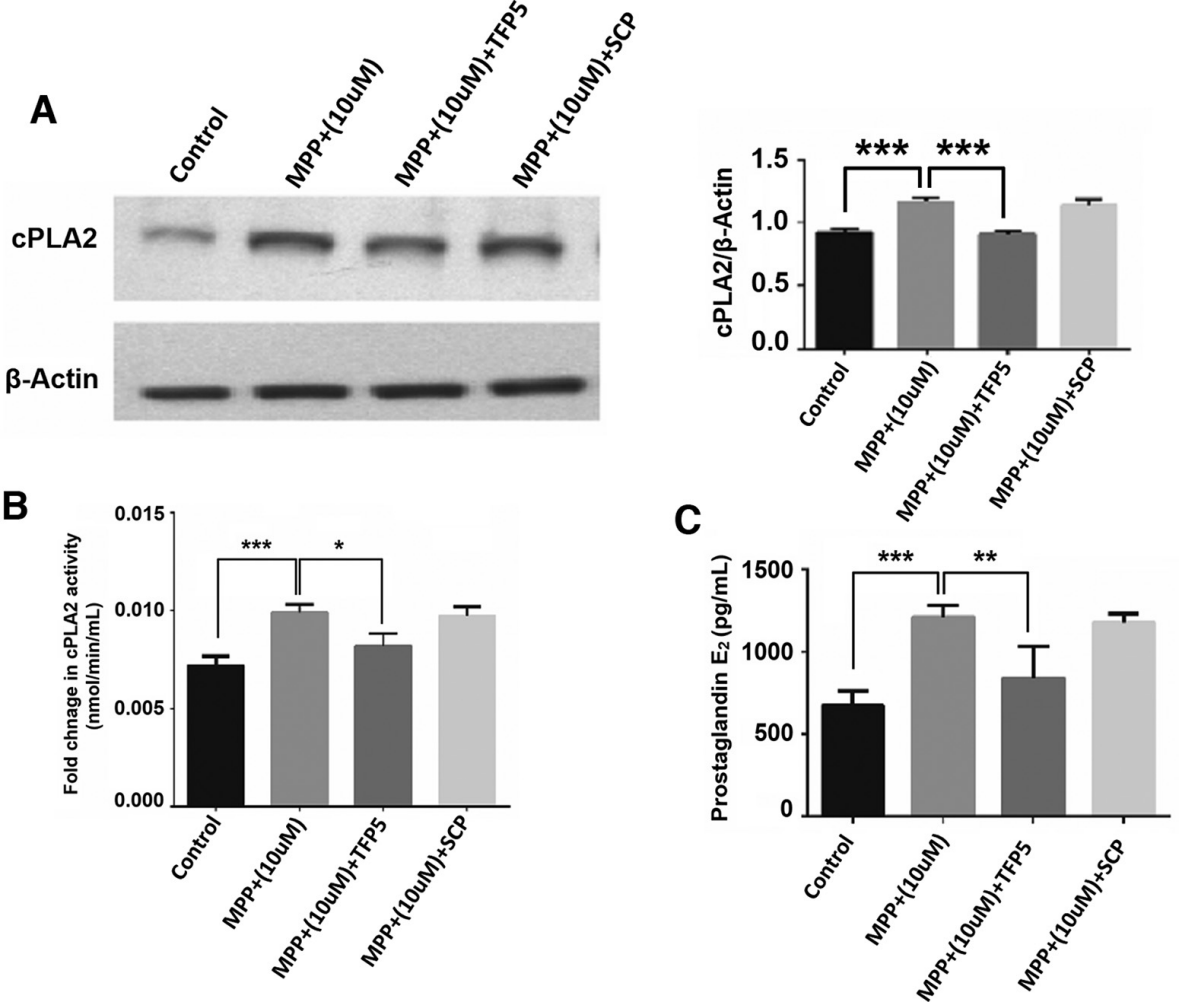
cPLA2 expression, its activity, and prostaglandin E2 synthesis in neuroglia cells with MPP^+^ and TFP5 treatment. ***A***, The blot showing cPLA2 expression pattern of control, MPP^+^ (10 μm), MPP^+^ (10 μm) + TFP5 and MPP^+^ (10 μm) + SCP in protein lysate of neuronal–glial culture along with its densitometric analysis. ***B***, The bar graph for the fold of change in cPLA2 activity of control versus MPP^+^ (10 μm) and MPP^+^ (10 μm) versus MPP^+^ (10 μm) + TFP5 in neuronal–glial culture. ***C***, The bar graph shows prostaglandin E2 amount in control versus MPP^+^ (10 μm) and MPP^+^ (10 μm) versus MPP^+^ (10 μm) + TFP5 in neuronal–glial culture. The MPP^+^ (10 μm) + SCP is used as a standard reaction for inhibitor treatment; *n* = 3. Data are presented as the mean ± SEM, and analysis was done using one-way ANOVA and Bonferroni’s test. *p < 0.05, **p < 0.01, ***p < 0.001.

It should be made clear that all experiments described in the pure astrocyte cultures were repeated with mixed neuronal–glial cultures to confirm that the inflammation cascade described is valid for neurons/glia. MPP^+^ treatment led to enhanced cPLA2 activity in the neuron–glia culture (Fig. 6*A*,*B*), and TFP5 treatment showed a decrease in the cPLA2 activity (Fig. 6*A*,*B*). Simultaneously, we also measured the PGE2 levels in mixed culture. We notice that similar to primary astrocytes cultures, enhanced PGE2 release in the MPP^+^-treated cells at the same time TFP5 treatment ameliorates the PGE2 release (Fig. 6*C*).

### Deregulated Cdk5/p25 activity in transgenic Parkinson’s disease mouse brain and activation of cPLA2

**Figure 7.**
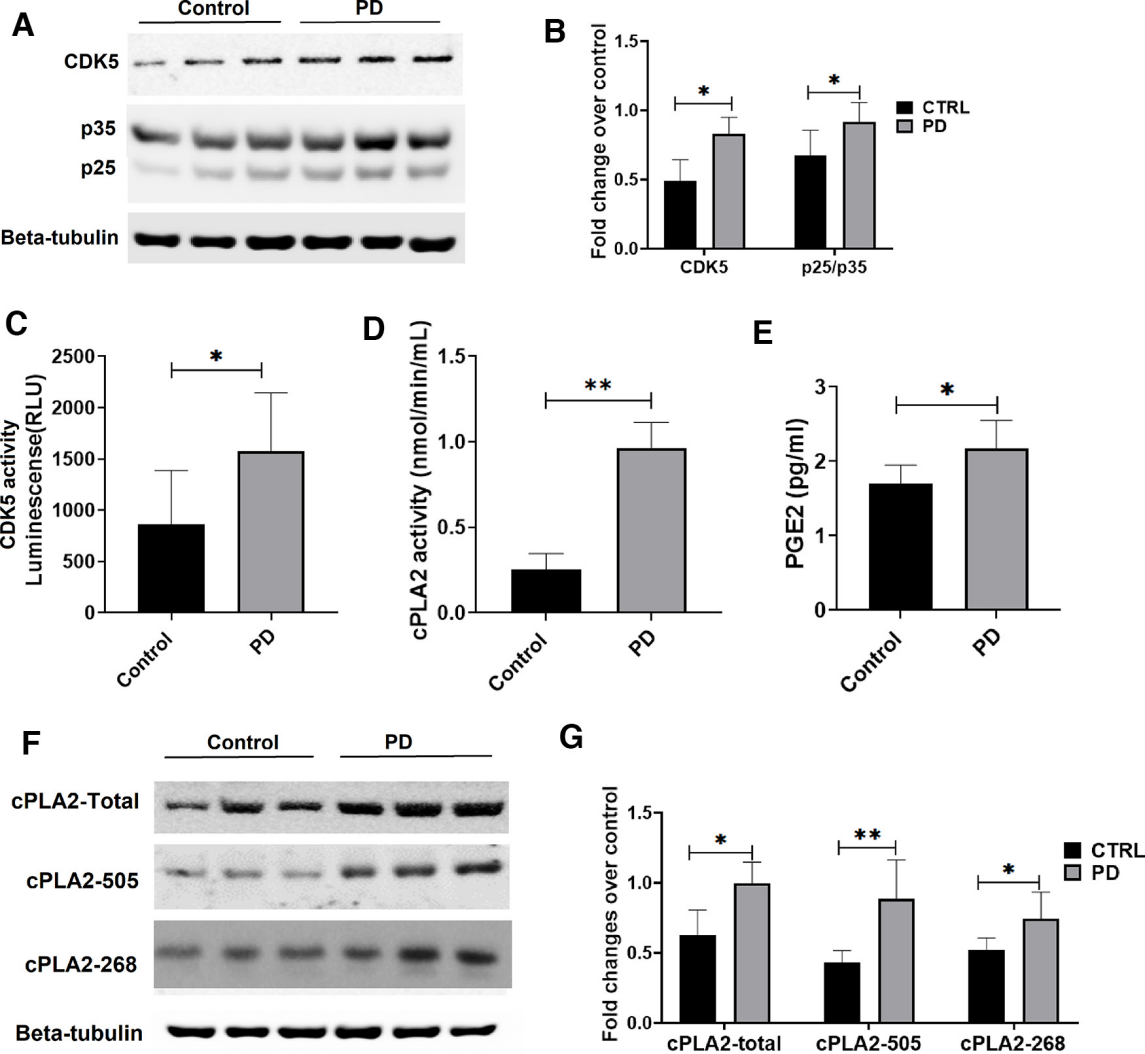
Generation of p25, Cdk5 activity, cPLA2 activity, and PGE2 levels along with the expression pattern of cPLA2 and phospho-cPLA2 in control versus PD mice model. ***A***, The blot shows the expression pattern of Cdk5, p35, and p25 in control versus PD mouse brain protein lysate (*n* = 3). ***B***, The densitometric analysis of Cdk5 expression and p25/p35 ratio over its standard, β-tubulin, in control versus PD mice (*n* = 6). ***C***, The bar graph shows Cdk5 activity that correlates with the luminescence (in relative light units) measure of control versus PD mouse brain protein lysate (*n* = 6). ***D***, The bar graph shows cPLA2 activity in the brain protein lysate of control versus PD mice model (*n* = 6). ***E***, The bar graph shows the PGE2 level (in picograms per milliliter) of control versus PD mice model. ***F***, Western blot analysis of cPLA2-total, cPLA2-505, and cPLA2-268 expression in control versus PD mice. ***G***, The bar graph shows fold changes of cPLA2-total, cPLA2-505, and cPLA2-268 expression over β-actin in control versus PD mice. Data are presented as the mean ± SEM, and analysis was performed using unpaired *t* test with Holm–Sidak method. *p < 0.05, **p < 0.01, ***p < 0.001.

The expression of p25 and p35 in the brain lysate of the PD mouse model was studied with respect to its control along with the Cdk5 activity. The result shows enhanced Cdk5 expressions along with the generation of p25 in the PD mouse brain compared with the control (Fig. 7*A*,*B*). Also, enhanced Cdk5 activity (Fig. 7*C*) supports dysregulation of Cdk5 by p25 in the PD brains. Since Cdk5/p25 is deregulated, we next checked the cPLA2 activity in the PD mouse brain. Figure 5*D* shows increased cPLA2 activity in the brains of PD mouse models compared with control mice. In addition, we could also see the increased level of prostaglandin E2 as well (Fig. 7*E*).

### Enhanced expression of cPLA2, Ser-505, and Thr-268 phosphorylation in PD mouse brain

**Figure 8.**
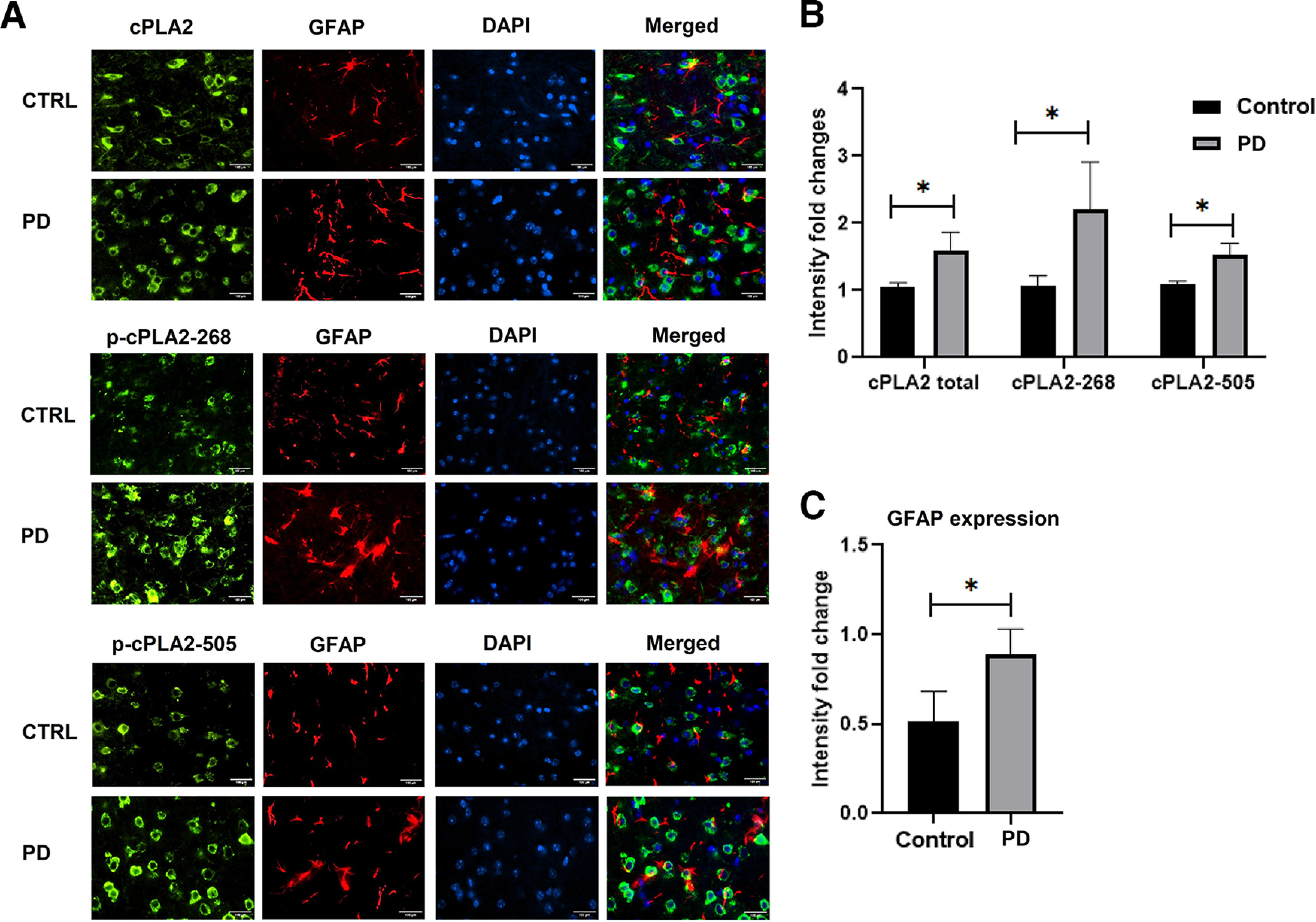
Astrocyte activation along with total and phospho-cPLA2 (S505 and T268) enhanced expression in the PD mouse brain. ***A***, Brain sections (10 μm) of control and PD mice were stained with GFAP, cPLA2 total, phospho-cPLA2 at T-268, and phospho-cPLA2 at S-505 in control versus PD mouse brain sections of the substantia nigra region. ***B***, GFAP immunodensity/mm^2^ in control versus PD (*n* = 3 mice/group). Scale bar, 100 μm. ***C***, cPLA2 and phospho-cPLA2 immunodensity/mm^2^ in control versus PD (*n* = 3 mice/group). All data are presented as the mean ± SEM, and analysis was performed using an unpaired *t* test and the Holm–Sidak method; *n* = 3. *p < 0.05, **p < 0.01, ***p < 0.001.

Since we found increased cPLA2 activity in transgenic PD mouse brain, we further checked the specific T-268 and S-505 phosphorylation levels. The custom made phospho-specific antibody for the site T-268 and commercial phospho-S505 antibodies are used. The brain protein lysate of Parkinson’s disease mice showed increased P-505 and P-268 compared with the respective control mice (Fig. 7*F*,*G*). In addition, we could see the activation of astrocytes, which corelates with enhanced GFAP expressions in the substantia nigra of PD brain model along with total cPLA2 and its phosphorylated forms (Fig. 8*A*,*B*). Immunohistochemistry studies also showed the increased expression of cPLA2 and its phosphorylated forms S505 and T268 in the substantia nigra region of the brains of PD mice compared with respective control mice (Fig. 8*A*,*C*).

### Human transcriptome analyses of Parkinson’s disease datasets

**Figure 9.**
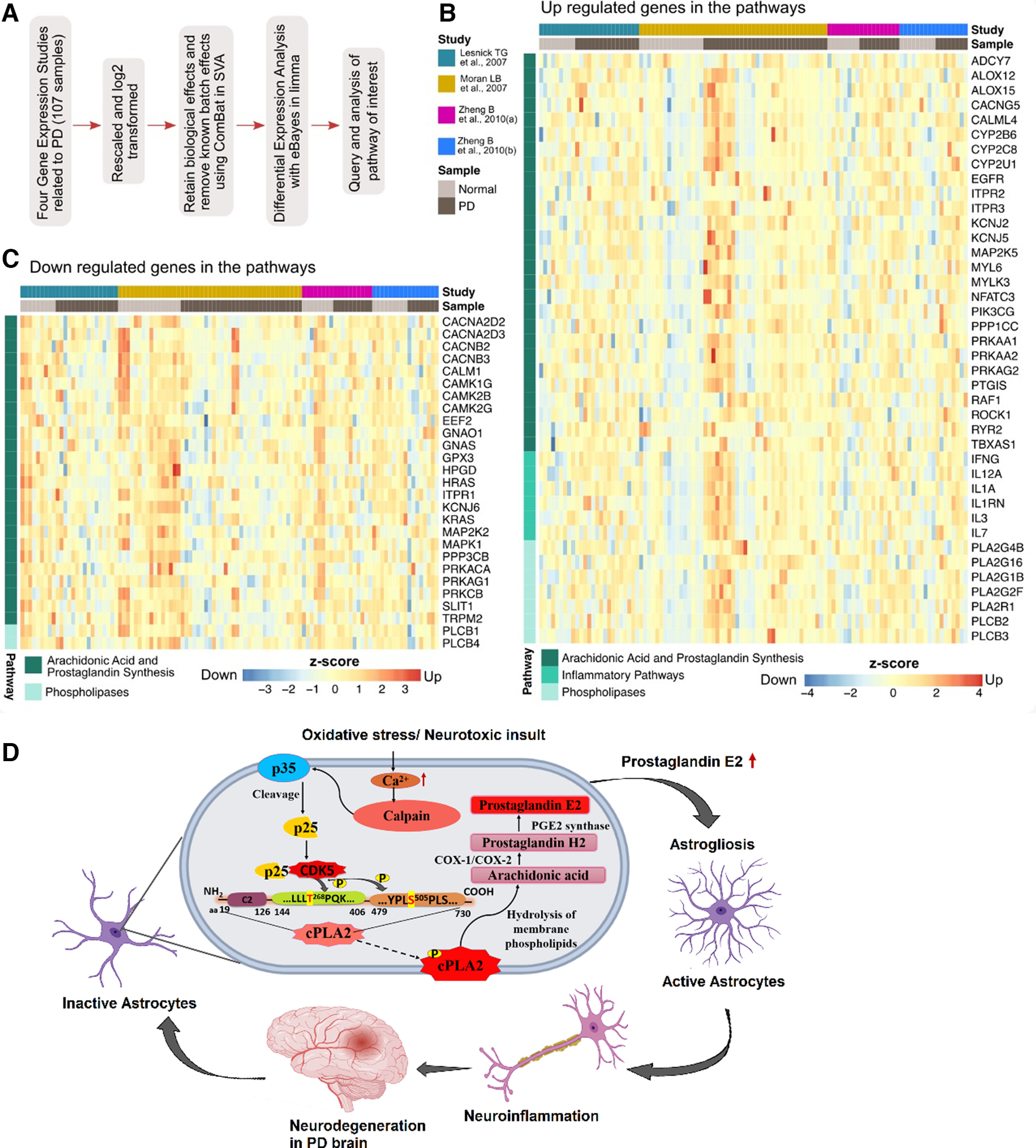
***A***, Workflow of the conjoint differential expression analysis of four PD transcriptomics datasets. ***B***, Heatmap showing upregulated genes in the arachidonic acid and prostaglandin synthesis and inflammatory pathways. ***C***, Heatmap showing downregulated genes in the AA and prostaglandin synthesis and inflammatory pathways. ***D***, The model proposed to explain the mechanism of Cdk5/p25-mediated cPLA2 activation that leads to neuroinflammation.

**Figure 10.**
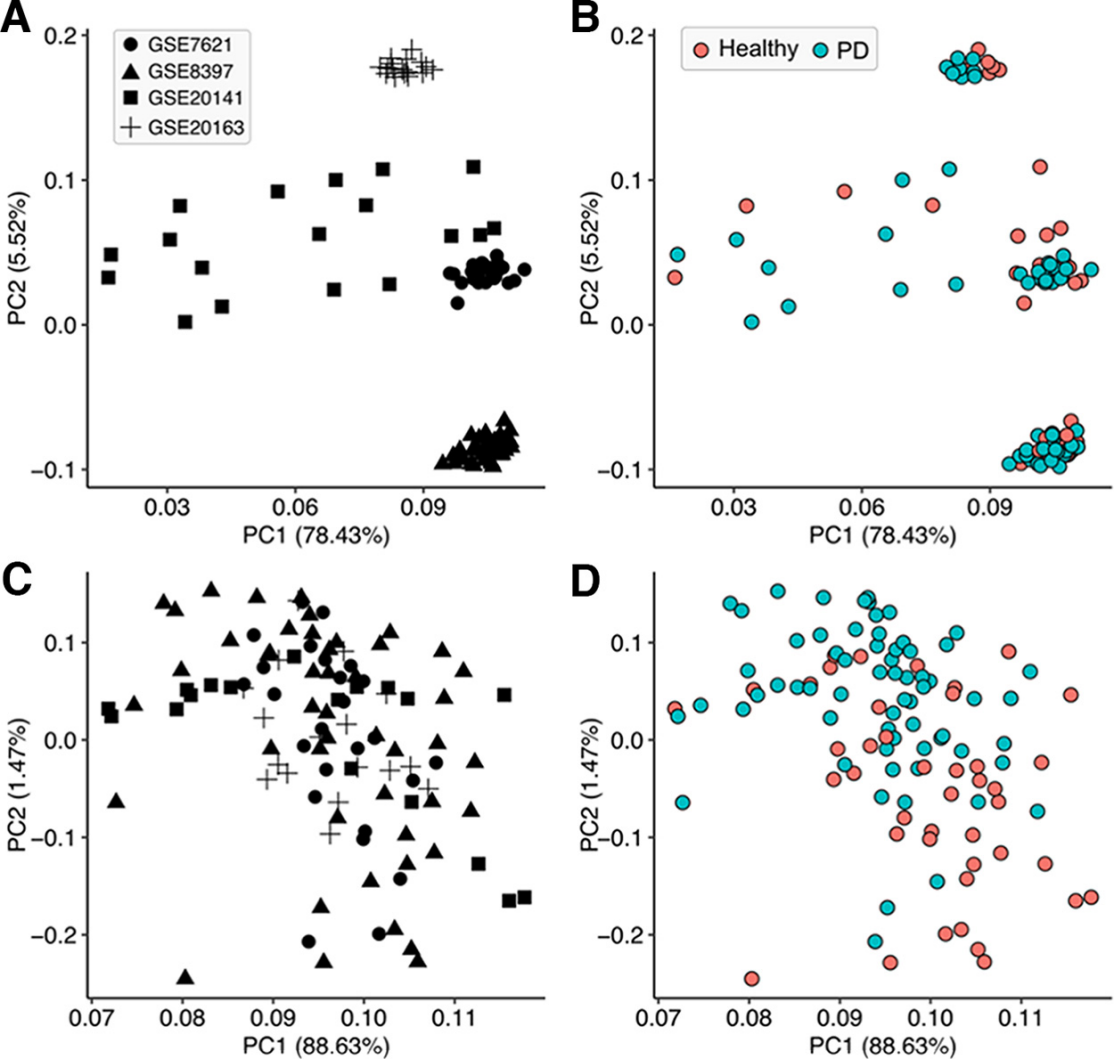
***A–D***, Principal component analysis without (***A***, ***B***) and with (***C***, ***D***) normalized expression profiles.

To understand whether neuroinflammation and other pathways such as prostaglandin and arachidonic acid synthesis are getting modulated in patients with PD, we performed the differential expression analysis of four independent datasets (107 samples) corresponding to substantia nigra of the postmortem samples of the PD (65) and healthy control (42) samples (Fig. 9*A*). We queried and performed further analysis with the list of genes of our pathways of interest (AA, prostaglandin, and inflammatory pathways). Of the list of 198 genes of the pathway of interest, 67 genes were found to be differentially expressed. Of this set of genes, 40 genes corresponding to the AA and prostaglandin synthesis metabolism in humans were significantly upregulated, whereas 27 genes were seen to be downregulated (Fig. 9*B*,*C*, respectively). The genes upregulated included various cytokines and inflammatory pathway genes including interleukins. Also, the various isoforms of phospholipases were seen to be upregulated. Figure 10 also provides the principal component analysis without and with the normalized expression profiles.

## Discussion

This study discloses new insights into the role of Cdk5 in the regulation of cPLA2 phosphorylation, activity, and membrane transport, and, finally, its implication in the PD mice. The individual single mutant of cPLA2 along with the double mutant fails to phosphorylate in the presence of active Cdk5/p35 and Cdk5/p25. However, the double mutant does not show an additively decreased level of phosphorylation. Again, in the presence of Cdk5 inhibitor TFP5, Cdk5 fails to phosphorylate cPLA2 wt (Fig. 1*A*). This confirms that S-505 and T-268 are the potential Cdk5 phosphorylation residues on cPLA2. We also checked whether these sites are important for cPLA2 kinase activity. These results are in agreement with the previous data. A phosphorylation-dependent increase in cPLA2 activity was observed in cell lysates derived from ATP-treated CHO cells or EGF-treated kidney mesangial cells (Gronich et al., 1988; Bonventre et al., 1990; Goldberg et al., 1990; Lin et al., 1992a,b). Moreover, it has been previously reported that MAPK can phosphorylate S-505 in cPLA2 and a mutation at the consensus site for MAPK in cPLA2 (S505A) fails to be phosphorylated or to generate the decreased electrophoretic mobility form of cPLA2 after incubation with MAPK *in vitro* (Lin et al., 1993). Through multiple ways, cPLA2 can be phosphorylated, and its activation has been regulated. Among these, intracellular Ca^2+^ has been shown to play an important role in the regulation of cPLA2. Multiple kinases including phorbol ester-activated protein kinase C, growth factor-activated receptor tyrosine kinases (Gronich et al., 1988; Bonventre et al., 1990; Goldberg et al., 1990; Lin et al., 1992a,b) and MNK1-related protein kinases (Hefner et al., 2000) have shown the phosphorylation and activation of cPLA2. In addition, a number of agents are also associated with increased serine phosphorylation of cPLA2 (Lin et al., 1992a). No data regarding the threonine phosphorylation of cPLA2 have been previously reported. We report here for the first time that Cdk5 phosphorylates T-268 position of cPLA2 in addition to S-505. Moreover, studies with cPLA2 lacking the consensus phosphorylation site for Cdk5, Ser-505, and Thr-268 indicate that Cdk5-mediated cPLA2 phosphorylation is essential for the kinase activity. Our MD studies also revealed large changes in the local structures of the mutant cPLA2 protein (Fig. 2). Moreover, structural distortions that we observed in our studies affect the stability of the global structure (Fig. 2*A*). Also changes in the microenvironment of the mutations that we could detect show an important role in protein destabilization (Fig. 2*B*). Our longer-timescale simulations have shown more significant structural changes, which could lead to the destabilization of the protein and affect its kinetic activity (Fig. 2*C*,*D*).

The membrane translocation of phosphorylated cPLA2 at Ser-505 and Thr-268 in the presence of Cdk5/p35 and Cdk5/p25, which was significantly decreased in case of single as well as double mutant cPLA2 (Fig. 3), was also shown. In the present study, we also reported that MPP^+^ stimulates Cdk5/p25 activation, enhances cPLA2 activity, labels AA release, and enhances prostaglandin E2 production from primary astrocytes culture along with neuroglia mixed culture (Figs. 5, 6). This is in agreement with previous studies that different agents, ATP, PMA, or A23187, could evoke the release of labeled AA from cells (Xue et al., 1999). In murine astrocytes, AA releases mainly because of cPLA2 activation, as Kennedy et al. (1995) reported that C57BL/6J mice are known to have a natural mutation causing a frameshift disruption in the group IIA sPLA2 gene (Kennedy et al., 1995). Our results agree with the notion that multiple phosphorylation sites are present in cPLA2 (de Carvalho et al., 1996; Gijón et al., 1999) and that different protein kinases may regulate its activity including Cdk5 (Börsch-Haubold et al., 1998; Geijsen et al., 2000; Gijón et al., 2000).

Phosphorylation of cPLA2 at S-505 by ERK1/2 and/or p38 MAPK has been shown to cause a shift in the electrophoretic mobility of cPLA2 (Lin et al., 1993; Kramer et al., 1996; Gijón et al., 2000) as well as an increase in enzyme activity. It has been also reported that homozygous mice with cPLA2 mutation were significantly resistant to MPTP-induced dopamine depletion compared with littermate control (Klivenyi et al., 1998). Activation of cPLA2 is observed under pathologic conditions where inflammation is present. Since accumulation and aggregation of α-synuclein (SN) is closely associated with PD pathogenesis (Lee and Trojanowski, 2006), the study also showed that α-SN induces synaptic damage through cPLA2 hyperactivation in α-SN-treated primary neurons (Bate and Williams, 2015). A previous study also reports neuroinflammation in the PD rat model through the upregulation of AA signaling mediated by cPLA2 (Lee et al., 2010). A recent study in PD patients reports a significant increase in serum lipoprotein-associated PLA2 (Lp-PLA2) compared with the control group. This shows a strong association of Lp-PLA2 with the risk of PD pathogenesis, and Lp-PLA2 serum levels can be used for the detection of PD (Wu et al., 2021). The finding of elevated levels of cPLA2 immunoreactivity in the PD brain supports the hypothesis that there is an active inflammatory process occurring in PD via cPLA2.

However, a previous report suggests that there is a close association between neurodegeneration and p25-mediated neuroinflammation (Muyllaert et al., 2008). Neuroinflammation and neurodegeneration are the main pathologic features of the PD brain. It was reported previously that the inhibition of Cdk5/p25 hyperactivation leads to reduced neurodegeneration in the PD mouse model (Binukumar et al., 2015). Last, we also checked whether Cdk5 hyperactivation takes place in the brain of transgenic PD mice. Here, we show the generation of p25 and hyperactivation of Cdk5/p25 in the brain of the transgenic PD mice model (Fig. 7*A–C*). We further checked cPLA2 kinase activity in the PD mouse brain. We observed a significant increase in the cPLA2 activity in brains of PD mice compared with those of control mice (Fig. 7*D*). Moreover, we also noticed hyperphosphorylation of cPLA2 at S-505 and T-268 residues in the brains of PD mice (Figs. 7*F*,*G*, 8*C–F*). We also observed the activation of astrocytes in the tissue sections of the PD mouse brain (Fig. 8*A*,*B*). Finally, activation of cPLA2 leads to a significant increase in the production of PGE2 levels (Fig. 7*E*) in the PD mouse brain compared with the control brain.

Our *in vitro* and *in vivo* results have pointed out that activated phospholipases induce the AA release and enhanced prostaglandin synthesis, and this is validated further with the conjoint transcriptomic analysis wherein significant upregulation in the AA, prostaglandin, and inflammatory pathways is noticeable in the case of PD patients compared with healthy control subjects (Fig. 9*B*). The levels of genes, such as PTGIS (prostaglandin synthase) and PLA2G (phospholipases), and inflammatory cytokines, such as IL-1A, IL-3, and IL-12, are higher in the PD patients. Upregulation in the oxytocin signaling pathway is also seen to increase the expression of cPLA2 and the calcium ion flux inside the cell (Soloff et al., 2000). Previous studies have shown that activated microglia contribute to the dopaminergic neuron degeneration in substantia nigra of PD patients (Caggiu et al., 2019; Karaaslan et al., 2021), and studies in murine PD models have also shown upregulation in AA signaling. Concordant with these findings, increased expression of genes encoding proinflammatory cytokines, and AA signaling and various other key regulators in these pathways have been found in our analyses. Figure 9*D* shows a proposed model to explain the mechanism of Cdk5/p25-mediated cPLA2 activation leading to neuroinflammation. Inhibiting the phosphorylation of S-505 and T-268 of cPLA2 or decreasing the deregulating Cdk5 kinase activity in humans could be a potential therapeutic intervention to rescue the excess neuroinflammation in the PD brain.

## Synthesis

Reviewing Editor: Karl Herrup, University of Pittsburgh School of Medicine Decisions are customarily a result of the Reviewing Editor and the peer reviewers coming together and discussing their recommendations until a consensus is reached. When revisions are invited, a fact-based synthesis statement explaining their decision and outlining what is needed to prepare a revision will be listed below. The following reviewer(s) agreed to reveal their identity: Bjoern Bauer. Note: If this manuscript was transferred from JNeurosci and a decision was made to accept the manuscript without peer review, a brief statement to this effect will instead be what is listed below.

The responses to the previous reviews have been comprehensive and have meaningfully improved the work.

## Author Response

Point-by-point reply to Referees

Synthesis Statement for Author (Required):

In this study, Paul et al. demonstrate CDK5/p25-mediated phosphorylation of cPLA2 at T-268 and S-505 leads to cPLA2 activation and translocation to the membrane, which increases AA generation and PGE2 production. The authors also demonstrate that MPP+ activates this mechanism, suggesting it could be involved in Parkinson’s disease. The authors also provide supporting data using a transgenic mouse Parkinson’s model. The study concludes with Parkinson’s disease patient transcriptome data analyses that support their findings in the animal model. Overall, this is a well-designed and carefully executed systematic study with a technically and methodologically sound approach that will be of intertest to the eNeuro readership. While the basics of the study are solid, there are important weaknesses that need to be corrected.

Response: We greatly thank the reviewer for their compliments about our work and its impact.

1.The most important of these is that the CDK5 kinase assays are not a direct measure of cPLA2 phosphorylation. The ADP-GloTM kit used measures the conversion of ADP to ATP, and the luminescence is indicative of ATP and consequent kinase activity. The graphs depicted for the kinase assays are correctly labeled as ‘CDK5 activity’. However, the authors interpret these results to be a measure of “decreased [cPLA2] phosphorylation” (e.g., line #287), and claim that CDK5 directly phosphorylates cPLA2 at S505 and T268. This conclusion is possible but is not directly proven by the data provided. To show that CDK5 does indeed phosphorylate cPLA2, mass spectrometry analysis or Western blot analysis (using the phosphorylation-specific cPLA2 antibodies) should be performed following the in vitro kinase assay. In addition, the purified in vitro assay in Fig. 1A there is no condition shown for CDK5/p25 or p35 alone, in the absence of cPLA2 wt. Thus, we do not know how much cPLA2 wt induces/increases CDK5 activity. This control needs to be shown.

Response: We thank the reviewer for these thoughtful comments and insightful suggestions. We completely agree with the reviewer’s statement that CDK5 kinase assay is not a direct measure of cPLA2 phosphorylation. Therefore, to strengthen our hypothesis we have performed a CDK5 kinase assay using in vitro synthesized pure cPLA2 wt and mutant proteins as a substrate along with CDK5/p35, and CDK5/p25 as kinases. After the kinase reaction, the products were examined in western blot using the phospho-specific cPLA2 at Ser-505 and Thr-268 (Figure 1B-E) antibodies.

Figure 1: cPLA2 phosphorylation by CDK5 (A) CDK5 kinase assay with cPLA2 wt and its mutants along with TFP5 treatment in vitro by its pure proteins. (B-E) Western blot images of phosphorylated cPLA2 i.e. p-cPLA2 268 and p-cPLA2 505 expressions after CDK5 kinase assay with cpla2 wt and its mutants as substrate. Total cPLA2 was considered as standard. (F) The Western blot image shows the expression of cPLA2 and its mutants post-transfection in HEK293T cells (G) Bar diagram showing CDK5 kinase activity with cPLA2 wt and mutant protein as substrates of CDK5 along with CDK5 inhibitor TFP5 (H) cPLA2 kinase activity: The bar graph shows cPLA2 activity of cPLA2 wt along with its three mutants S505A, T268A and DM, also a comparative analysis of its mutants with the cPLA2 wt activity. Data are presented as mean {plus minus} S.E.M and analysis was done using one-way ANOVA and Bonferroni test; n=3, *p<0.05, **p<0.01, ***p<0.001.

2. The results shown in figures 3C and D seem to be at odds with the text which claims that cPLA2 is phosphorylated by CDK5. The blots illustrated show no apparent change in the levels of phosphorylated S268 or T505 cPLA2 when CDK5/p25 or CDK5/p35 is overexpressed neither in the membrane nor the cytosolic fractions. They were not quantified and, at least on the surface, seemingly contradict the claim that CDK5 directly phosphorylates cPLA2 at S505 and T268. In conjunction with the points raised above, the authors should replicate and quantify these data.

Response: Thank you for your suggestions. The blots in Figure 3C and D shows changes in phosphorylated S505 and T268 in membrane vs cytosolic fraction. To clarify the results we have quantified the data and added a graph of relative expression in the membrane as well as the cytosolic fraction of p-cPLA2 268 and cPLA2 505 with respect to total cPLA2 (Figure 3E and 3F) in the revised version of the manuscript.

Figure 3: The expression of cPLA2 wt and its mutants in the membrane and cytosolic protein fraction (A)The expression pattern of cPLA2 and its mutants in a membrane and cytosolic fraction of HEK293T protein lysate co-transfected with cPLA2 and its mutants along with CDK5/p35 and CDK5/p25. The reaction standard used for membrane fraction is calnexin and for cytosolic fraction is beta-tubulin. (B) Cytosolic and membrane fraction fold changes over control. (C) Membrane translocation of phosS268 cPLA2 in the presence of active CDK5 Kinase. (D) Membrane translocation of phosT505 cPLA2 in the presence of active CDK5 Kinase (E) Densitometry of relative expressions of p-cPLA2-268 in cytosolic and membrane fractions. (F) Densitometry of p-cPLA2-505 in cytosolic and membrane fractions. Calnexin is used as membrane fraction control and beta-tubulin as cytosolic frantic control. Data are presented as mean {plus minus} S.E.M and analysis was done using a one-way ANOVA test followed by a Bonferroni post hoc; n=3, *p<0.05, **p<0.01, ***p<0.001. ,***p<0.001

3. To more convincingly show that CDK5 drives membrane translocation of cPLA2, the authors should include controls using the CDK5 inhibitor (TFP5) in Fig 3. While the results are consistent with the hypothesis, overexpression of CDK5 may activate other kinases (e.g., MAPK) leading to indirect effects. CDK5 inhibitor experiment will clarify this result. It is curious that the authors did not use phospho-specific antibodies to support the membrane translocation studies. One important additional control would be to include a total cPLA2 lane in the membrane fraction for the T268 site studies (Fig. 3C).

Response: We agree with the referee. We have now included the results of CDK5/p25 inhibitor (TFP5) in Figures 3C and 3D which strengthens the fact that CDK5 drives the translocation of cPLA2 in the revised manuscript. We have also used phospho-specific antibodies both p-cPLA2-268 and p-cPLA2-505 in figures 3C and 3D respectively.

Figure 3: The expression of cPLA2 wt and its mutants in the membrane and cytosolic protein fraction (A)The expression pattern of cPLA2 and its mutants in a membrane and cytosolic fraction of HEK293T protein lysate co-transfected with cPLA2 and its mutants along with CDK5/p35 and CDK5/p25. The reaction standard used for membrane fraction is calnexin and for cytosolic fraction is beta-tubulin. (B) Cytosolic and membrane fraction fold changes over control. (C) Membrane translocation of phosS268 cPLA2 in the presence of active CDK5 Kinase. (D) Membrane translocation of phosT505 cPLA2 in the presence of active CDK5 Kinase (E) Densitometry of relative expressions of p-cPLA2-268 in cytosolic and membrane fractions. (F) Densitometry of p-cPLA2-505 in cytosolic andmembrane fractions. Calnexin is used as membrane fraction control and beta-tubulin as cytosolic frantic control. Data are presented as mean {plus minus} S.E.M and analysis was done using a one-way ANOVA test followed by a Bonferroni post hoc; n=3, *p<0.05, **p<0.01, ***p<0.001. ,***p<0.001.

4.In addition to these concerns there are technical issues that should be addressed. First, it would appear that western blots throughout the manuscript have been re-probed, raising concerns about their reliability. If this is the case, this should be stated in the Methods, and key experiments should be validated with independent blots to increase the confidence in the findings.

Response: The phospho specific antibody probed blots for p-cPLA2-268 and p-cPLA2-505 were reprobed with the total cPLA2 antibody. All the other blots used in the manuscript are not reprobed but the membranes were cut at different positions with the help of its ladder for probing with different antibodies. This statement now incorporated into the revised manuscript

5.The claims of increased cPLA2 in the nigra of the Parkinson’s disease model mice should clarify whether the intensity was normalized by total cell number (preferably). The authors might also wish to replace their chosen examples with images that more clearly support the claim of increased cPLA2. In this vein, the immunostaining appears to be localized to neurons. A cell type marker such as GFAP or RALDH1 to demonstrate astrocytic localization would help.

Response: We agree with the suggestion. The intensities of all the immunohistochemistry images were normalized by the total cell number i.e intensity of DAPI. We have mentioned the same in our revised version. We have now also tried to localize total-cPLA2 as well as phospho-cPLA2 with GFAP in the sections as per reviewer’s recommendation. Revised figure 8 now shows colocalization and enhanced expression of cPLA2 along with GFAP in the PD brain compared to control.

Figure 8: Astrocyte activation along with whole and phospho-cPLA2 (S505 and T268) enhanced expression in the PD mouse brain. (A) Brain sections (10 μm) of control and PD mice were stained with GFAP in the control vs PD mice brain section of the substantia nigra region. (B) GFAP immunodensity/mm2 in control vs PD (n =3 mice/group) (C)Expression of GFAP in control vs PD mice brain section (D) Expression of phospho cPLA2 at S-505 (E) Expression of phospho cPLA2 at T-268 in control vs PD mice brain section. Scale bar: 200 μm. (E) cPLA2 and phospho-cPLA2 immunodensity/mm2 in control vs PD(n =3 mice/group). All data are presented as mean {plus minus} S.E.M and analysis unpaired t-test and Holm Sidak method ; n=3, *p<0.05, **p<0.01, ***p<0.001. ,***p<0.001

6. The authors must provide antibody concentrations, not just the dilutions. In keeping with journal policy, provide more information such as the product number for the respective vendors for all the materials in the Methods.

Response: We apologize for not providing complete information about the antibody concentrations as well as product numbers. We have mentioned all the details of antibodies used in the materials and methods section of the revised manuscript.

7. There are statistical issues that need attention. The authors use a one-way ANOVA test followed by a Bonferroni post hoc test for their analysis, but this may not be applicable for many analyses throughout the paper such as Fig. 3B, Fig 7B-G, Fig, 8B and F. For most of these results, a t-test is should have been performed. Please also indicate the test used in each figure legend and if statistical significance is claimed in the text or the legend, please ensure that it is shown on the figure itself.

Response: We apologize for the confusion. Proper details have been added in the materials and methods part. The results with only two groups present in Fig. 3B, Fig 7B-G, Fig, 8B and F were analyzed with unpaired t-test using the Holm Sidak method and all the other analyses were one-way ANOVA tests followed by a Bonferroni post hoc test. We have indicated the test performed for all the analyses in each figure legends respectively along with statistical significance in the revised manuscript.

Other minor issues:

7.Line #350 cites Fig 3A while results refer to Fig3 C and D. Please correct.

Response: We greatly thank the reviewer for catching our error. It has been corrected in the revised Manuscript. We have given proper care to rectify all the minor mistakes regarding Figure no. and proper labeling of the figures in the revised manuscript.

8.The data shown in Figure 5C should be quantified as there is concern that the claims in the text do not match the data in the figure.

Response: We apologize for not being able to explain figure 5 properly in the text previously. We have modified the text according to our results in Figure 5 in the revised version.

9.The MPP+ data in Figure 5 should be normalized to total protein levels.

Response: It has been corrected in the revised manuscript.

9.Molecular weight standards should be labeled

Response: It has been corrected in the revised manuscript.

10.The label of “cpm” should be defined.

Response: It has been corrected in the revised manuscript.

11.Figure 1B should show the result of the co-immunoprecipitation (bound and unbound fractions) and not just the input expression levels given that the immunoprecipitation samples were used for the CDK5 kinase assay.

Response: It has been corrected in the revised manuscript.

12.It would be better if calnexin and β-tubulin were probed in membrane and cytosolic fractions as another test of the fractionation procedure.

Response: The membrane and the cytosolic fraction controls used in the manuscript were Calnixin and beta tubulin respectively. The same is also mentioned in the revised manuscript (Figure 3 legend)

13. In Figure 4, clarify the antibodies used for IP and for blotting.

Response: The antibodies used for immunoprecipitation as well as antibodies used for western blot probing are now mentioned clearly the the Figure 4B

Figure 4: Colocalization of CDK5 and cPLA2 (A) Colocalization of CDK5 and cPLA2.

Immunocytochemistry analysis of CDK5 and cPLA2 showing their expressions and colocalization. (B) Western blot data of CDK5 and cPLA2 pull-down IPs developed with cPLA2 and CDK5 antibodies respectively.

14.The blot in Figure 7F is of poor quality and should be replaced and the results requantified.

Response: The blots in Figure 7F have been replaced with new figure and the results were again requantified and presented in the revised version of the manuscript.

Figure 5: Generation of p25, CDK5 activity, cPLA2 activity and PGE2 levels along with expression pattern of cPLA2 and phospho-cPLA2 in Control vs PD mice model (A)The blot shows the expression pattern of CDK5, p35 and p25 in Control vs PD mice brain protein lysate (n=3). (B) The densitometric analysis of CDK5 expression and p25/p35 ratio over its standard, beta-tubulin, in control vs PD mice (n=6). (C) The bar graph shows CDK5 activity which correlates with the luminescence(RLU) measure of control vs PD mice brain protein lysate (n=6) (D) The bar graph shows cPLA2 activity in the brain protein lysate of Control vs PD mice model (n=6). (E) The bar graph shows the PGE2 level in pg/ml of control vs PD mice model (F)Western blot analysis of cPLA2-total, cPLA2-505 and cPLA2-268 expression in Control vs PD mice (G) The bar graph shows fold changes of cPLA2-total, cPLA2-505 and cPLA2-268 expression over beta-actin in control vs PD mice.Data are presented as mean {plus minus} S.E.M and analysis was done using unpaired t-test with Holm Sidak method ; *p<0.05, **p<0.01, ***p<0.001. ,***p<0.001.
